# DNA demethylation and tri-methylation of H3K4 at the *TACSTD2* promoter are complementary players for TROP2 regulation in colorectal cancer cells

**DOI:** 10.1038/s41598-024-52437-1

**Published:** 2024-02-01

**Authors:** A. Gehring, K. Huebner, H. Rani, K. Erlenbach-Wuensch, S. Merkel, V. Mahadevan, R. Grutzmann, A. Hartmann, R. Schneider-Stock

**Affiliations:** 1 Experimental Tumorpathology, Universitätsklinikum Erlangen, FAU Erlangen-Nürnberg, Erlangen, Germany; 2 Institute of Pathology, Universitätsklinikum Erlangen, FAU Erlangen-Nürnberg, Erlangen, Germany; 3 Institute of Bioinformatics and Applied Biotechnology (IBAB), Bangalore, India; 4 Department of Surgery, Universitätsklinikum Erlangen, FAU Erlangen-Nürnberg, Erlangen, Germany; 5 Comprehensive Cancer Center Erlangen-EMN (CCC ER-EMN), Bavarian Cancer Research Center (BZKF), Erlangen, Germany; 6 FAU Profile Center Immunomedicine (FAU I-MED), FAU Erlangen-Nürnberg, Erlangen, Germany

**Keywords:** Cancer, Computational biology and bioinformatics, Molecular biology, Molecular medicine

## Abstract

TROP2 is a powerful cancer driver in colorectal cancer cells. Divergent epigenetic regulation mechanisms for the corresponding *TACSTD2* gene exist such as miRNAs or DNA methylation. However, the role of *TACSTD2* promoter hypermethylation in colorectal cancer has not been investigated yet. In this study, TROP2 expression strongly correlated with promoter methylation in different colorectal tumor cell lines. Treatment with 5-Azacytidine, a DNMT1 inhibitor, led to demethylation of the *TACSTD2* promoter accompanied by an increase in TROP2 protein expression. TROP2 expression correlated with promoter methylation in vivo in human colon tumor tissue, thereby verifying promoter methylation as an important factor in the regulation of TROP2 expression in colorectal cancer. When performing a ChIP-Seq analysis in HCT116 and HT29 cells, we found that *TACSTD2* promoter demethylation was accompanied by tri-methylation of H3K4. In silico analysis of GSE156613 data set confirmed that a higher binding of histone mark H3K4me3 around the *TACSTD2* promoter was found in *TACSTD2* high expressing tumors of colon cancer patients compared to the corresponding adjacent tumor tissue. Moreover, the link between TROP2 and the H3K4me3 code was even evident in tumors showing high intratumoral heterogeneity for TROP2 staining. Our data provide novel evidence for promoter demethylation and simultaneous gains of the active histone mark H3K4me3 across CpG-rich sequences, both being complementary mechanisms in the transcriptional regulation of *TACSTD2* in colon cancer. The functional consequences of TROP2 loss in colorectal cancer needs to be further investigated.

## Introduction

Colorectal cancer is one of the most frequent and deadliest cancer types worldwide^1^. Colorectal carcinogenesis is determined by an accumulation of genetic and epigenetic alterations that are closely correlated with tumor histology, tumor progression, and metastasis. In addition, there is enormous intratumoral heterogeneity that is not least caused by epigenetic alterations such as promoter methylation or histone modifications^2,3^. Promoter methylation is an epigenetic modification affecting CpG dinucleotides in the promoter region of genes^3^. Cytosines are methylated to 5-methylcytosine through DNA methyltransferase enzymes such as DNMT1, DNMT3A, and DNMT3B. While DNMT3A and DNMT3B are de novo DNA methyltransferases, DNMT1 is a replication-dependent DNA methyltransferase^3^. In general, hypermethylation of promoter regions leads to gene silencing^4^. In addition, the active histone mark H3K4me3 seems to further enrich the accessibility of DNA to transcriptional machinery^5^.

The so-called CpG island methylator phenotype (CIMP) constitutes a colorectal cancer subtype characterized by an extraordinary high frequency of methylated genes. It is hypothesized that clonal selection for methylated genes leads to highly aggressive subclones that are the precursors for invasion and metastasis and thus difficult to detect and treat. Moreover, methylated genes might generate a predisposition platform for specific cancer-related mutations. Indeed, CIMP-low and CIMP-high distinct sets of tumors are associated with specific epigenotypes (p53, K-Ras, BRAF)^6^. Therefore, it is no surprise that epigenetic alterations, especially DNA promoter methylation, are discussed as biomarkers for early detection, prognosis or therapy response^7,8^.

Trophoblast cell surface antigen 2 (TROP2) is often overexpressed in colorectal cancer^9^. TROP2 is a transmembrane glycoprotein encoded by the intronless *TACSTD2* gene on chromosome 1 at gene locus 1p32^10^. *TACSTD2* has a CpG island upstream of the transcriptional start site with a GC content above 50% and a ratio of CpG dinucleotides above 0.6^11^. Although the functions of TROP2 overexpression are well studied and are associated with tumor aggressiveness, EMT, metastasis and decreased overall survival^9,12,13^, little is known about the regulation of *TACSTD2* expression. Among the known transcription factors modulating *TACSTD2* mRNA expression are NFκB, ATF2, CREB1 and different members of the GRHL family^11,14^. A recent study described a novel RNA epigenetic mechanism affecting TROP2 translation in bladder cancer^15^. There are a few studies showing miRNA-based *TACSTD2* regulation^16,17^. TROP2 loss by promoter methylation has already been reported for different cancer types, such as tamoxifen-resistant breast cancer^18^, lung adenocarcinoma^19^, cholangiocarcinoma^20^, renal cell cancer^21^ and hepatocellular carcinoma^22^. However, whether this epigenetic regulation principle also applies for colorectal cancer remains unknown.

Thus, we aimed to analyse whether *TACSTD2* expression might be silenced by promoter methylation in colorectal cancer and further explored if binding of the active H3K4me3 code could facilitate *TACSTD2* gene transcription.

## Materials and methods

### Cell lines and cell culture

For the experiments, the following human colorectal cancer cell lines were used: DLD1, LoVo, LS-174T, SW620, SW480, SW837, HT29, and HCT116. The origins of these cell lines are given in Supplementary Table 1. All cell lines were genotyped using Multiplex Cell Authentication by Multiplexion (Heidelberg, Germany) and were regularly tested negative for mycoplasma contamination.

Except for SW620 all cell lines were cultured in RPMI 1640 with stable glutamine and 2.0 g/L NaHCO3 (PAN Biotech, Aidenbach, Germany) supplemented with 10% fetal bovine serum (FBS) (PAN-Biotech) and 1% penicillin‒streptomycin (P/S; PAN-Biotech). The cell line SW620 was cultured in DMEM (Gibco/Life Technologies) supplemented with 10% FBS and 1% P/S. The cells were cultured in a humidified atmosphere at 37 °C and 5% CO_2_. For the collection of cell pellets, cells were seeded at specific cell numbers (1.5 × 10^6^ for HCT116 and HT29; 3.0 × 10^6^ for DLD1 and LoVo; 2.5 × 10^6^ for LS-174T and SW480; 4.0 × 10^6^ for SW837; 7.0 × 10^6^ for SW620) and collected after 48 h.

### Tumor specimens

33 cases of Formalin-fixed paraffin-embedded (FFPE) tissue samples were used; 13 tumor tissues paired with adjacent tissue, 10 tumors paired with non-tumor (non-adjacent) tissues, 8 only normal (non-adjacent) tissue and 2 tumor tissue paired with adjacent and normal (non-adjacent) tissue. All samples were collected at the University Hospital in Erlangen, Germany. The requirement for formal ethics approval was exempted by ethics committee of the Universitätsklinikum of the Friedrich-Alexander Universität Erlangen-Nürnberg. All procedures were performed in accordance with Declaration of Helsinki. According to the ethics committee written informed consent of patients was waived by ethics committee of the Universitätsklinikum of the Friedrich-Alexander Universität Erlangen-Nürnberg (24.01.2005, 18.01.2012, 04.09.2012) in this retrospective study since all clinical data were used completely anonymously. Clinical data on age, sex, diagnosis, tumor histology and tumor stage can be found in the supplemental materials (Supplementary Table 2).

### Western blot analysis

Preparation of cell lysates was performed as previously described^14^. Briefly, 35 µg of protein was separated by SDS-polyacrylamide gel electrophoresis (SDS-PAGE). After protein transfer onto nitrocellulose membranes and blocking for nonspecific binding, the membranes were incubated with different primary antibodies at 4 °C overnight. Antibody details are given in Supplementary Table 3. Signals were detected with the Immobilon Western Chemiluminescent HRP substrate kit (Merck Millipore, Burlington, MA, USA) and analysed with the GeneGnome imaging system (Syngene, Bangalore, India). For quantification of Western blots, band intensities of proteins of interest were quantified by ImageJ (National Institute of Health, USA) and normalized to each corresponding GAPDH (housekeeper). Ratios for cleaved PARP were determined by ImageJ, but ratios were calculated by dividing cleaved PARP by non-cleaved PARP.

### DNA isolation

#### From cell pellets

Genomic DNA was isolated from cell pellets using NucleoSpin^®^ Tissue (Macherey-Nagel, Düren, Germany) following the manufacturer’s instructions. DNA was quantified using a NanoDrop™ ND-1000 spectrophotometer (Thermo Fischer Scientific Waltham, MA USA).

#### From FFPE tissue samples

Genomic DNA was isolated from six slices of 5 µM thick FFPE human samples from marked tumor, adjacent non-tumor and normal (non-adjacent) areas using the QIAamp DNA FFPE Tissue Kit (Qiagen, Hilden, Germany) following the manufacturer’s instructions. The areas were marked in the HE-stained slices in consultation with a pathologist (KEW). The DNA concentration was quantified using a NanoDrop™ ND-1000 spectrophotometer.

### Pyrosequencing

Using the EZ DNA Methylation-Gold Kit (Zymo Research, Irvine CA, USA), 500 ng of genomic DNA from cells and FFPE tissue samples was modified by bisulfite conversion. As described in the user’s manual and in Kreutz et al.^23^, PCR was performed with the PyroMark PCR Kit using 2 µl of the bisulfite-converted DNA. The PyroMark CpG Assay PCR primer set (Qiagen) and three self-designed primer pairs using PyroMark Assay Design Software were used. Primer sequences can be found in Supplementary Fig. 1. The PCR product was prepared for pyrosequencing using the PyroMark vacuum prep tool (Qiagen). Using the corresponding sequencing primers for our four primer pairs, the samples were analysed by PyroMark Q24 (Qiagen). The results were evaluated using PyroMark Q24 Software (Qiagen). In total, 21 CpG dinucleotides were analysed for all DNAs extracted from cells. For the DNA isolated from human tissue slices, CpG Primer1 could not be used, resulting in 17 analysed CpG sites. 10 cases were exemplarily analysed for all 17 sites, for the majority only 6 CpG Dinucleotides were analysed using the PyroMark CpG Assay PCR primer set. The details for the whole sequence with all CpG dinucleotides can be found in the Supplementary materials (Supplementary Fig. 1).

### Methylation-specific polymerase chain reaction (MSP)

In addition, methylated and unmethylated CpG sites in the *TACSTD2* promoter region were detected using two different sets of primer pairs for PCR analysis. For the methylated sequence, the primers were M-forward: 5′-GTTATTTAAATATTAGTGGGGACGG-3′ and M-reverse: 5′-ATAATAAAACGAAAAAACGCGAA-3′, and for the unmethylated sequence, the primers were U-forward: 5′-TGTTATTTAAATATTAGTGGGGATGG-3′ and U-reverse: 5′-CCATAATAAAACAAAAAAACACAAA-3′. The methylated PCR product had a length of 188 bp and the unmethylated PCR product had a length of 191 bp. The PCR mix consisted of 12.5 µl of 10 × HotStarTaq Master Mix (Qiagen), 6.5 µl ddH_2_O, 2.5 µl of 10 µM of each primer and 1 µM of bisulfite converted DNA. PCR was carried out in a thermocycler, starting with 15 min at 95 °C, then 36 cycles (denaturation for 30 s at 95 °C, annealing for 60 s at 58 °C and extension for 30 s at 72 °C) followed by 10 min at 72 °C. Then, 6 × loading dye was added to the PCR product, which was separated by a 2% agarose gel, stained with Roti-Safe (Carl Roth, Karlsruhe, Germany) and visualized under UV illumination.

### 5-Azacytidine treatment

5-Azacytidine was purchased from Sigma-Aldrich (St. Louis, MO, USA) and was prepared as described in the user’s manual. Twenty-four hours after seeding, HCT116, HT29, LoVo, and LS-174T cells were treated with the DNMT1 inhibitor 5-Azacytidine at different concentrations (1 µM, 5 µM and 10 µM). Forty-eight hours after the start of treatment, the cells were retreated again for an additional 24 h. After a total of 72 h, the treatment was stopped. For HT29 and HCT116 cells, the pellets were collected for Western blot and DNA analysis. LoVo and LS-174T cells were seeded again, and after 72 h of recovery, cell pellets were collected for Western blot and DNA analysis.

### Immunohistochemistry staining

FFPE colorectal tumor samples were obtained from excision biopsies as described previously^14^. Tissue blocks were assembled. Slices (2 µM thick) were stained with HE, TROP2 (1:2000, rabbit IgG, ab214488, Abcam, Cambridge, UK), and H3K4me3 (1:1000, rabbit IgG, #9751, Cell Signaling, Danvers, USA). The samples were scored for TROP2 expression by a pathologist (KEW). The total immunostaining score (0–300) consists of the intensity (0–3) and the percentage of positive cells (0–100%). Using the median of 90, the tumor samples were categorized into a TROP2 high-expressing group (> 90) and a TROP2 low-expressing group (< 90).

### ChIP-Seq analysis

ChIP-Seq was performed for the H3K4me3 mark in HT29 and HCT116 cells. QC the adapter content, Phred score and GC content in all th samples were assessed by FastQC (version0.11.7). Reads were later mapped to the human genome (hg38) using Bowtie2 (version 2.4.1). Sam files were converted into sorted and indexed BAM files using SAM tools (version 1.9). MACS2 (version 2.2.6) was used for each replicate separately. Bigwig tracks of H3K4me3 were normalized using RPKM and a bin size of 10. Heatmaps were generated using plotHeatmap. To normalize the raw counts and to identify differential peaks (absolute log2-fold-change > 1 and FDR < 0.01 using the Wald test) in the two technical replicates of each condition DEseq2 (version 3.6.0) was used. The gained and lost sites were annotated using annotatePeaks.pl and hg38 as a reference genome. The representative genome browser snapshot is rendered using IGV. All ChIP-Seq data were downloaded from the GEO database under the accession number GSE143653.

###  RNA-Seq data processing

RNA-Seq data used in this study are available in the GEO database under accession number GSE156613^24^. A total of six CRC patients, for which both RNA and ChIP-Seq data of H3K4me3 were available, was used for analysis. For 3 of them also the adjacent non-tumor tissue was analysed. Details of patient data are given in Supplementary Table 4. The normalized FPKM values were downloaded for further analysis. The median values were used to divide each cohort into high and low *TACSTD2* expression groups.

###  ChIP-Seq analysis

The ChIP-Seq data utilised for this study are available in the GEO database under accession number GSE156614^24^. FastQC  (version 0.11.7) was extracted for all samples to check for adapter content, Phred score and GC content. The adaptor sequence was removed using Cutadapt (version 2.6) to clean ChIP-Seq raw data. Cleaned reads were mapped into the human reference genome (hg38) using BWA (version 0.7.17) with default settings. Peak calling was performed using MACS2 For each tumor patient, the corresponding input sample was used for background normalization. To compare different ChIP-Seq data sets, peaks obtained in each condition (tumor, adjacent tumor tissue) were merged using the “merge” function from bedtools. The density of reads in each merged region was quantified using normalized signal per million reads. Gained and Lost sites were defined on the basis of normalized signals per million reads of H3K4me3 for which fold change (FC) was larger than > 1 between low and high *TACSTD2* expressing group. Bigwig tracks of H3K4me3 were normalized using CPM and binsize of 1. Heatmaps were generated using plotHeatmap. The information about the distance to the nearest promoter provided by Homer after the annotation was used to interpret the peaks as promoters (± 2000 bp of the TSS). The representative genome browser snapshot was shown using IGV.

### Statistical analysis

For statistics, GraphPad Prism v. 8.3.0 (GraphPad, San Diego, CA, USA) software was used. p values were determined using Mann–Whitney test and Wilcoxon test. Statistical significance was considered for p < 0.05.

## Results

### TROP2 expression strongly correlated with promoter methylation in colorectal tumor cell lines

**Figure 1:**
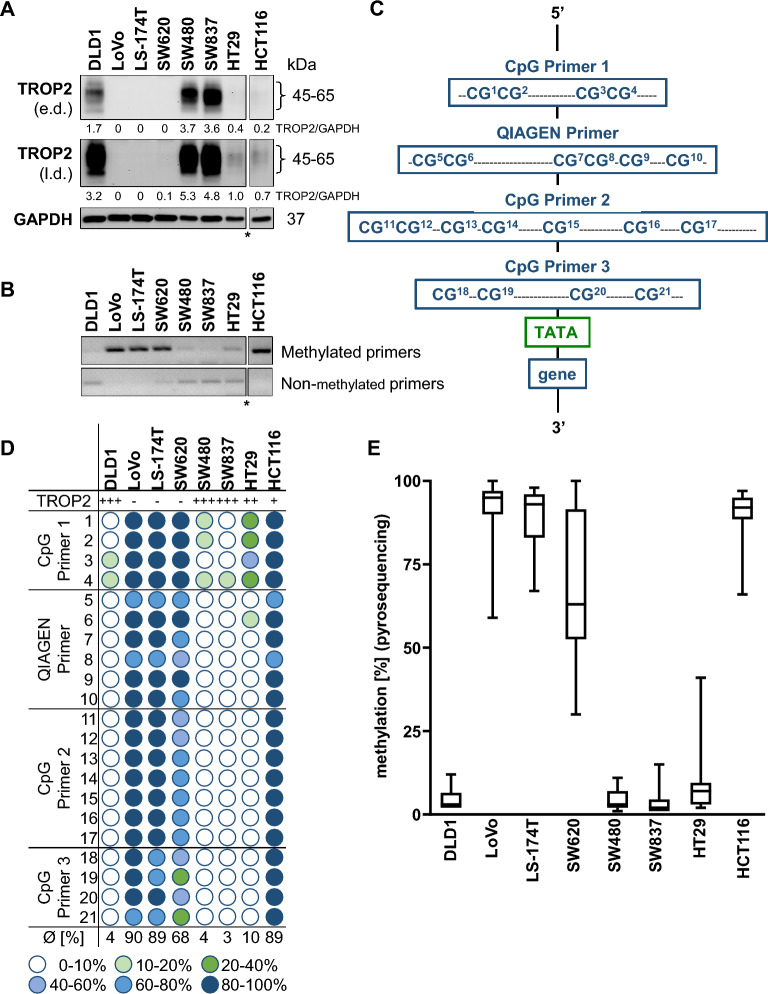
Expression level of TROP2 and methylation status of the *TACSTD2* promoter in different CRC cell lines. (**A**) TROP2 expression levels detected by WB for 8 different CRC cell lines, *all cell lines blotted onto the same membrane, lanes have been spliced out, e.d. = early development, l.d. = late development. Band intensities were quantified using ImageJ analysis software and ratios were calculated against the GAPDH band intensity. (**B**) MSP analysis for all cell lines using methylated and non-methylated primer pairs, *all cell lines were loaded onto the same gel, bands have been spliced out. (**C**) Schematic representation of the *TACSTD2* promoter with primer sequences for pyrosequencing covering 21 CpG dinucleotides. (**D**) Methylation status of CpG dinucleotides in the *TACSTD2* promoter for 8 different CRC cell lines. (**E**) The average methylation status for all cell lines detected by pyrosequencing.

To identify the relationship between *TACSTD2* promoter hypermethylation and TROP2 protein expression, we first determined the TROP2 expression status in seven different colon cancer cell lines (DLD1, LoVo, LS-174T, SW480, HT29, SW620, and HCT116) and one rectal cancer cell line (SW837) by western blotting. We identified a very heterogeneous pattern in TROP2-high and TROP2-low cell lines (Fig. 1A). Next, we analysed the methylation status at the *TACSTD2* promoter region by MSP analysis (Fig. 1B) and pyrosequencing (Fig. 1C–E; Supplementary Fig. 1). We found a clear inverse correlation between promoter methylation and protein expression. Heavily methylated cell lines LoVo, LS-174T, SW620 and HCT116 showed the lowest TROP2 expression, whereas low methylated DLD1, SW480 and SW837 cells had the highest TROP2 expression. HT29 cells showed moderate TROP2 expression with correspondingly partly methylated CpGs (Fig. 1A,B,D,E). Strikingly, there was high heterogeneity among the single CpGs (Fig. 1D).

### 5-Azacytidine treatment led to demethylation of the *TACSTD2* promoter accompanied by an increase in TROP2 protein expression

**Figure 2:**
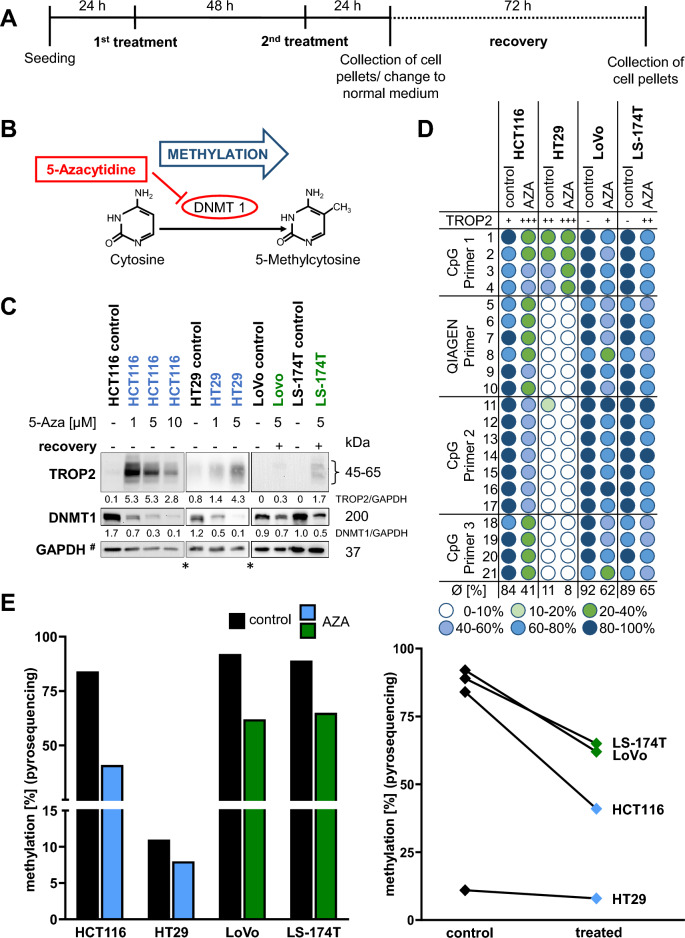
Inhibition of methylation with DNMT1i treatment in TROP2 low-expressing cell lines. (**A**) Schematic workflow in cell culture of DNMT1i treatment over 72 h with optional addition of 72 h recovery. (**B**) 5-Azacytidine inhibits methylation of cytosine to 5-methylcytosine via inhibition of DNA methyltransferase enzyme 1. (**C**) WB of TROP2 expression after treatment with DNMT1i in TROP2 low/no-expressing cell lines, blue-marked cell lines with 72 h of 5-Azacytidine treatment, green-marked cell lines with additional 72 h of recovery added to the treatment, ^#^same GAPDH blot was used in Suppl. Fig. 2 for HCT116 cells, *all cell lines were loaded onto the same gel, bands have been spliced out. Band intensities were quantified using ImageJ analysis software, and ratios were calculated against the GAPDH band intensity. (**D**) Methylation status in the *TACSTD2* promoter after DNMT1i treatment, as detected by pyrosequencing. (**E**) Average decrease in methylation in all CpG dinucleotides by DNMT1i. Cell lines are marked with green and blue as described for (**C**).

To address whether TROP2 expression is dependent on *TACSTD2* promoter methylation, TROP2 low-expressing cell lines (HCT116, LoVo, and LS-174T) and the moderate-expressing cell line HT29 were treated with the DNMT1 inhibitor 5-Azacytidine (5-Aza) at three different concentrations (Fig. 2A,B). 5-Aza exposure decreased the levels of DNMT1 in a dose-dependent manner (Fig. 2C). A concentration of 10 µM was proven to be highly toxic (Supplementary Fig. 2A). The TROP2 expression status after 5-Aza treatment was determined by western blotting and compared to untreated control cells. For all cell lines, a clear demethylation of approximately 25% was seen in pyrosequencing (Fig. 2D,E). 5-Aza treatment for 72 h led to re-expression of TROP2 in HT29 and HCT116 cells (Fig. 2C). For LoVo and LS-174T cells 72 h 5-Aza treatment did not increase TROP2 expression and an additional recovery phase was needed to increase the demethylating effect of 5-Aza (Fig. 2C; Supplementary Fig. 2B).

Overall, these results indicated that TROP2 expression is regulated by DNA methylation in colon cancer cells.

### TROP2 expression correlated with promoter methylation in vivo

**Figure 3:**
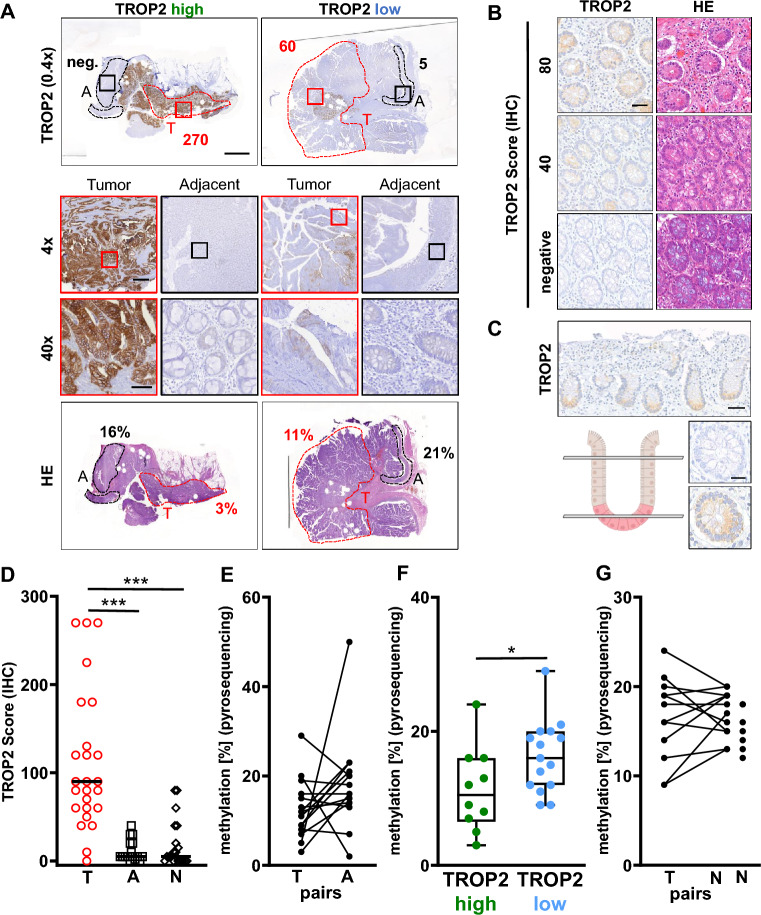
Comparison of primary tumor, adjacent non-tumor and normal (non-adjacent) samples regarding their TROP2 expression and *TACSTD2* promoter methylation. (**A**) Exemplary TROP2 IHC and HE staining for a TROP2 high and a low expressing tumor case, A = adjacent non-tumor (black), T = tumor (red), marked sections match the scratched tumor and adjacent non-tumor areas for the assay, TROP2 score from IHC staining given in the TROP2 stained slices, percentage in HEs shows average *TACSTD2* promoter methylation status for the different areas, scale bar in overview pictures equals 4000 µm, in the 4 × magnification 400 µm and in 40 × magnification 60 µm. (**B**) TROP2 expression in normal (non-adjacent) tissue with corresponding HE staining, scale bar 20 µm. (**C**) Localisation of TROP2 expression at the bottom of the colon crypts in normal tissue, scale bars 50 µm (overview) and 20 µm (smaller pictures), scoring is dependent on cutting depth. (**D**) Tumor, adjacent non-tumor and normal (non-adjacent, = N) sections compared for their TROP2 score determined by IHC staining, ***(T to A) p < 0.0001 (Mann–Whitney test; n(T) = 25, n(A) = 16), ***(T to N) p < 0.0001 (Mann–Whitney test; n(N) = 20). (**E**) Tumor and adjacent non-tumor sections compared for their average *TACSTD2* promoter methylation, p = 0.1307 (Wilcoxon test, n = 15). (**F**) TROP2 high-expressing tumor (TROP2 score > 90) and TROP2 low-expressing tumor (TROP2 score < 90) groups compared for their average *TACSTD2* promoter methylation *p = 0.0452 (Mann–Whitney test, n = 25). (**G**) Tumor and normal (non-adjacent) sections compared for their average *TACSTD2* promoter methylation (n = 12) and methylation of extra normal tissue samples (n = 8).

To further confirm our results in vivo, 15 pairs of corresponding colorectal cancer tumor and adjacent non-tumor tissue samples were evaluated regarding their TROP2 expression and *TACSTD2* promoter methylation status. When evaluating the TROP2 score based on immunohistochemical (IHC) staining (Fig. 3A), a significantly higher TROP2 score was observed in the tumor samples compared to adjacent non-tumor samples and normal tissue (Fig. 3B–D). These results are in line with previous studies showing overexpression of TROP2 in various cancer types, including colorectal cancer^9^. Next, DNA was isolated from FFPE tissue slices with marked tumor and adjacent non-tumor areas (exemplary cases are shown in Supplementary Figs. 3, 4). Remarkably, comparing the methylation status in tumor and adjacent non-tumor areas, 9 cases showed an increase in methylation in the adjacent non-tumor tissue; 5 cases had even lower methylation, and one case showed the same methylation level (Fig. 3E, Supplementary Fig. 5). This might reflect very early epigenetic alterations in the immediate vicinity of the tumor that are still invisible under the microscope. Next, the tumors were divided into two groups: TROP2 high and TROP2 low expressing tumors using the median of the TROP2 immunostaining score. Here, an inverse correlation between promoter methylation and TROP2 expression was seen with TROP2 high-expressing tumors (TROP2 score > 90) showing significantly less methylation than TROP2 low-expressing tumors (TROP2 score < 90, Fig. 3F). In addition, 12 tumor/normal (non-adjacent) pairs and 8 cases with only normal tissues were analysed. When comparing normal (non-adjacent) tissue with tumor tissue we found 6 of 12 cases with higher methylation, one case showing the same methylation level and 5 cases having lower methylation (Fig. 3G). As reported by Švec et al.^25^ TROP2 expression in normal tissue was mainly localized at the bottom of the colon crypts (Fig. 3C). Thus, the moderate TROP2 positivity in a few cases of normal tissue might be caused by different cutting depths at the crypt–villus axis (Fig. 3B,C). This could explain the high variation in TROP2 expression in normal tissues and the lack of correlation with the methylation status. In summary, our data suggest an inverse correlation between *TACSTD2* promoter methylation and TROP2 expression in colorectal cancer in vitro and in vivo.

### *TACSTD2* promoter demethylation is accompanied by methylation of H3K4me3

**Figure 4:**
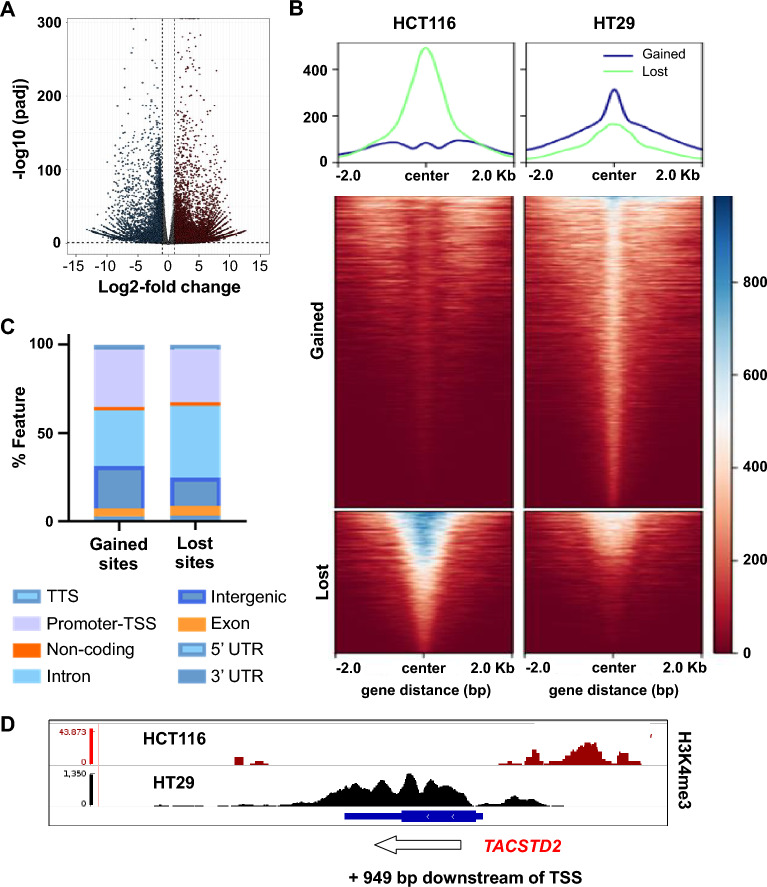
ChIP-Seq for active histone mark H3K4me3. (**A**) Volcano plot representing differential binding analysis of H3K4me3 ChIP-Seq in HCT116 cells compared to HT29 cells. Lost sites are represented in blue (8864), while gained sites are represented in red (11,511). (**B**) Heatmaps of normalized H3K4me3 ChIP-Seq read densities centered at the midpoints (± 2 kb) of 11,511 gained and 8864 lost regions. Each row represents a single region. The profile plots (top panel) show the average ChIP signals of gained (blue) and lost (green) sites. Signal intensity profile of the H3K4me3 mark in HCT116 and HT29 cells around gained and lost H3K4me3 sites are presented. (**C**) Stacked bar plot representing the genomic distribution of the identified gained and lost sites in HT29 cells compared to HCT116 cells. (**D**) IGV screenshot of H3K4me3 ChIP-Seq tracks at the *TACSTD2* locus in HCT116 (red) and HT29 (black) cells. The Y-axis represents normalized read counts of H3K4me3 within the locus of *TACTD2* (X-axis), and the direction of transcription is indicated by arrowheads.

DNA hypomethylation does not necessarily mean that a promoter is completely transcriptionally active^26^. To further promote transcription, novel active epigenetic marks such as H3K4me3 are gained at hypomethylated but still transcriptionally inactive CpG rich DNAs. To test this mechanism for the *TACSTD2* promoter, a ChIP-Seq analysis for the active H3K4me3 mark in HCT116 and HT29 cells was evaluated (Supplementary Fig. 6). A differential binding analysis was performed using DESeq2. The analysis revealed that when comparing HT29 cells with HCT116 cells, a total of 11,511 peaks were significantly gained and 8864 were lost in HT29 cells (FDR < 0.05, log2FC|1|), as represented by a volcano plot (Fig. 4A). The identified gained and lost sites were plotted as heatmaps with a flanking window of ± 2000 bp from the center of the peaks, as represented in the heatmap (Fig. 4B). This suggests that there was an overall increase in the active H3K4me3 mark in HT29 cells compared to that in HCT116 cells. The genome-wide distribution of the identified gained and lost sites suggested differential enrichment of H3K4me3 marks around intergenic regions and intronic regions (Fig. 4C). Gained sites showed higher occupancy around the intergenic region (24%) compared to lost sites (16%), while the lost sites showed higher occupancy around the intronic region (40%) compared to gained sites (31%) (Fig. 4C). Next, the *TACSTD2* promoter was examined for the H3K4me3 mark. Promoters were defined as those regions that are ± 2000 bp of the transcription start site (TSS). The results are documented in the displayed IGV browser tracks at the *TACSTD2* locus (Fig. 4D). Enrichment of the H3K4me3 histone mark was found at the HT29 *TACSTD2* promoter (+ 949 bp downstream from the promoter-TSS), but to a significantly lesser extent at the *TACSTD2* promoter of HCT116 cells (Fig. 4D).

### High *TACSTD2* expression correlates with elevated H3K4me3 binding at its promoter in CRC patients

**Figure 5:**
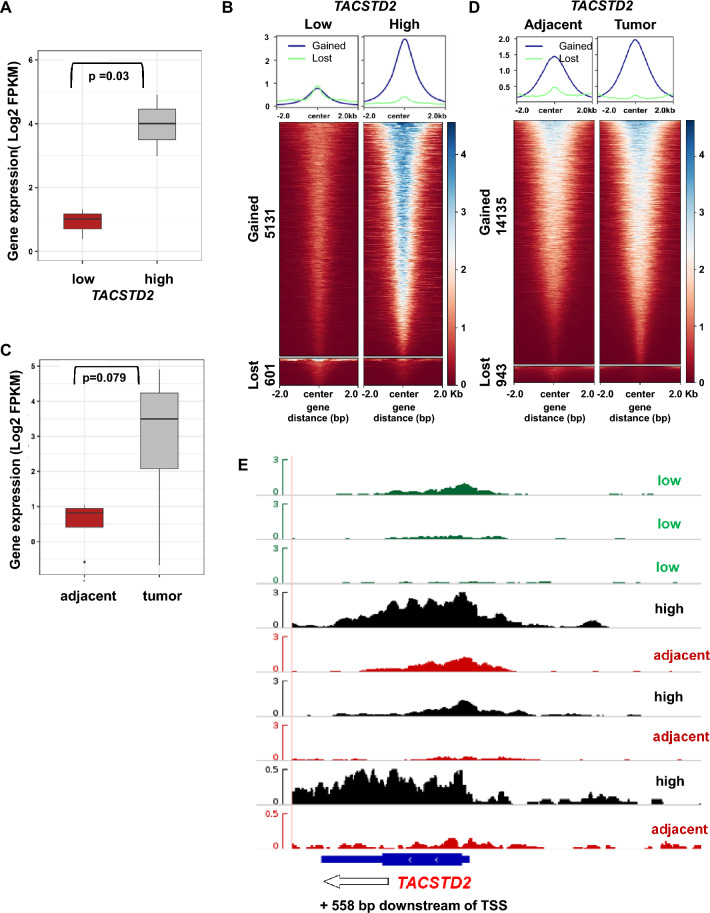
In silico validation of *TACSTD2*/H3K4me3 association in CRC patients through RNA and ChIP-Seq. (**A**) Box plot representing the differential *TACSTD2* gene expression profile in colorectal cancer patients (p value < 0.03). (**B**) Heatmaps of normalized H3K4me3 ChIP-Seq read densities centered at the midpoints (± 2 kb) of 5131 gained and 601 lost regions. Each row represents a single region. The profile plots (top panel) show the average ChIP signals of gained (blue) and lost (green) sites. Signal intensity profile of the H3K4me3 mark in low and high *TACSTD2* expressing CRC around gained and lost H3K4me3 sites are presented. (**C**) Box plot representing the differential expression distribution profile of *TACSTD2* between adjacent non-tumor and tumor tissues (p value < 0.079). (**D**) Heatmaps of normalized H3K4me3 ChIP-Seq read densities centered at the midpoints (± 2 kb) of 14,135 gained and 943 lost regions. Each row represents a single region. The profile plots (top panel) show the average ChIP signals of gained (blue) and lost (green) sites. Signal intensity profile of the H3K4me3 mark in normal and tumor CRC patients around gained and lost H3K4me3 sites are presented. (**E**) IGV screenshot of H3K4me3 ChIP-Seq tracks at the *TACSTD2* locus in low *TACSTD2* expressing tumors (green), *TACSTD2* high expressing tumors (black) and corresponding adjacent non-tumor tissue (red). The Y-axis represents normalized read counts of H3K4me3 within the locus of *TACSTD2* (X-axis), and the direction of transcription is indicated by arrowheads.please enlarge this figure and adapt to size of figure 4!!!!!!

To validate the association between high *TACSTD2* expression and elevated levels of H3K4me3 at the *TACSTD2* promoter in colorectal cancer patients, first the expression values of *TACSTD2* in 6 CRC patients was evaluated for which both, ChIP and RNA-Seq data, were available (GSE156613^24^). The cohort was divided into two groups representing low and high expressing *TACSTD2*, based on the median FPKM value (Fig. 5A). Next, differential binding analysis was performed based on the normalized signal values and identified 5131 gained H3K4me3 sites in the *TACSTD2* high expressing cohort compared to the *TACSTD2* low expressing cohort with only 601 enriched H3K4me3 sites. The identified gained and lost sites were plotted as heatmaps with a flanking window of ± 2000 bp from the center of the peaks as shown in the heatmap (Fig. 5B). Next, the *TACSTD2* expression of tumor and adjacent non-tumor tissues were analysed (Fig. 5C). The Box plot confirmed higher *TACSTD2* expression in tumor tissues. Interestingly, also an elevated association of the active H3K4me3 histone mark was identified at the promoters of the tumor tissues with 14,135 gains as compared to the adjacent non-tumor tissue with only 943 gains (Fig. 5D). The changes in H3K4 methylation at the *TACSTD2* locus were given in the displayed IGV browser tracks confirming a clear gain in H3K4me3 marks at the promoter regions of *TACSTD2* high-expressing tumors (Fig. 5E). Finally, this analysis was performed for three pairs of tumor and corresponding adjacent non-tumor tissue with high *TACSTD2* expression. As expected, there was an enrichment of the H3K4me3 code at the *TACSTD2* promoter of tumor tissue when compared to adjacent non-tumor tissue (Fig. 5E). This confirmed that elevated levels of active H3K4me3 marks at the promoter of the *TACSTD2* locus correlated with higher *TACSTD2* expression in CRC patients. Thus, our data suggest that promoter demethylation and simultaneous gains of the active histone mark H3K4me3 across CpG-rich sequences are complementary mechanisms in the gene transcription regulation of *TACSTD2* in colon cancer tissues.

### High TROP2 expression correlates with elevated H3K4me3 staining in colon tumors

**Figure 6:**
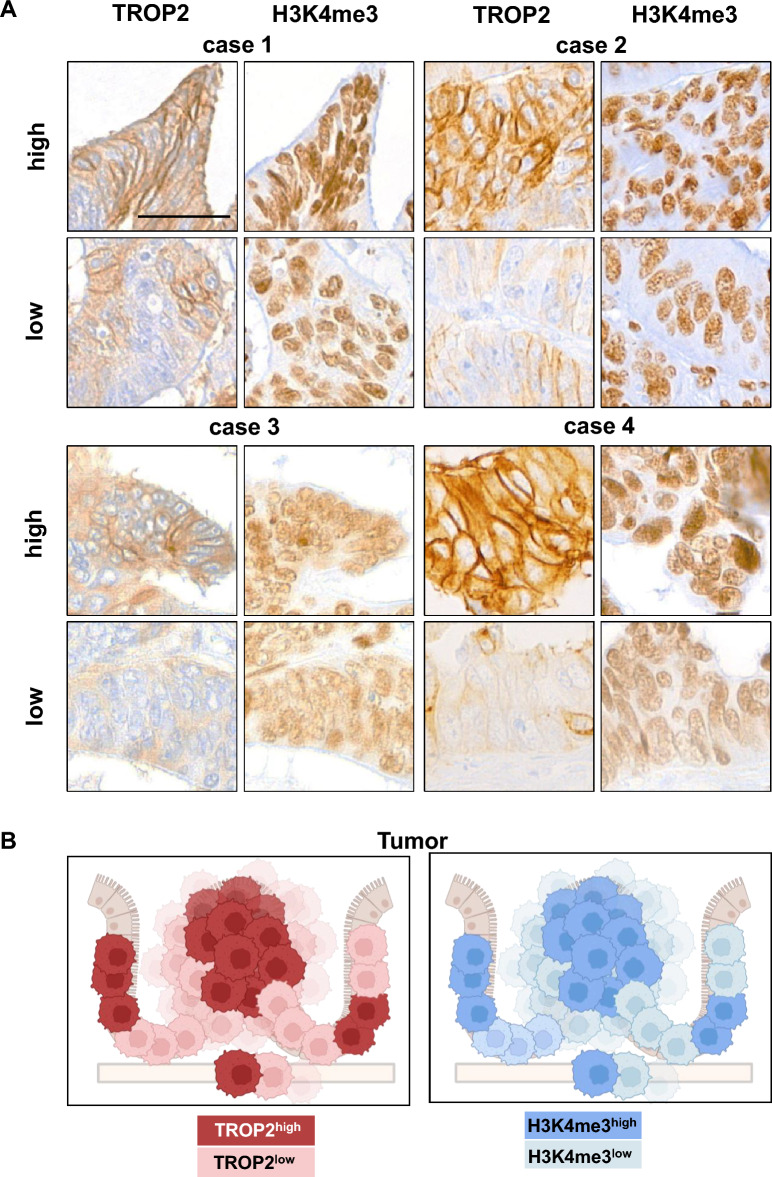
Comparison of TROP2 and H3K4me3 staining in colon tumors. (**A**) High and low TROP2 expressing sections of 4 exemplary tumor cases with intratumoral heterogeneity in TROP2 expression compared for TROP2 and H3K4me3 IHC staining, scale bar 40 µm. (**B**) Working model for correlation between TROP2 and H3K4me3 expression in colon tumors.

Next, we aimed to analyse the link between high TROP2 expression and high active H3K4me3 mark in colon tumors using immunohistochemistry. We identified 4 cases with high but remarkably heterogeneous TROP2 expression pattern (Supplementary Fig. 7). Here, tumor areas with high TROP2 expression were alternating with areas with no or low TROP2 expression. When evaluating H3K4me3 staining in such heterogeneous cases, we found a clear link between both stainings (Fig. 6A). Tumor areas with high TROP2 expression showed homogeneously a strong nuclear H3K4me3 signal whereas tumor areas with low/no TROP2 expression showed a rather heterogeneous staining pattern for H3K4me3 with mostly weakly positive stained nuclei. These findings suggest a positive link between *TACSTD2* promoter demethylation, high H3K4me3 binding, and high *TACSTD2* expression. This suggestion was summarized in the working model shown in Fig. 6B.

## Discussion

In concert, both DNA methylation and histone modifications are able to shape the expression pattern of genes in tumorigenesis, thereby remarkably expanding the regulatory capacity of the genome^3,27^. A deeper understanding of the epigenetic regulation principles for colon cancer driver genes will help at least to improve diagnosis but also to develop new prognostic and therapeutic options for this aggressive tumor type. TROP2 seems to be a cancer gene chameleon showing tumor suppressor or oncogene functions dependent on experimental stimulus and tumor type. There are many levels of regulation for TROP2 in colon cancer^28^, but so far, nothing is known about *TACSTD2* promoter hypermethylation. We found an inverse correlation between DNA methylation of the *TACSTD2* promoter and TROP2 mRNA/protein expression in colon cancer cells and human colon cancer samples. Treatment with the DNMT1 inhibitor 5-Azacytidine attenuated gene silencing in different colon cancer cell lines. We discovered an additional level of regulation with H3K4me3 triggering *TACSTD2* expression in cases with hypomethylated gene promoters.

Patient adjacent non-tumor and normal (non-adjacent) tissues showed significantly lower TROP2 levels and overall the TROP2 expression correlated with promoter methylation. The differences were not so clear for the adjacent non-tumor tissue. Whereas the majority of the cases had higher methylation in the adjacent non-tumor tissue compared to the corresponding tumor tissue, we also found lower or equal methylation levels. We suggest two hypotheses to explain these findings. First, the tissue surrounding the tumor might already be preneoplastic regarding early epigenetic alterations, and second, TROP2 might be downregulated at the tumor border/invasion front, as recently shown for oral squamous cell carcinoma^29^. Nevertheless, there are also TROP2-negative or low expressing tumors that are heavily methylated.

The consequences of TROP2 loss in cancer seem to be highly divergent. In liver cholangiocarcinoma and renal cell carcinoma, *TACSTD2* silencing by promoter hypermethylation was associated with enhanced aggressiveness properties such as proliferation, migration, metastasis or advanced tumor stage^20,21^. In contrast, in metastatic colon cancer patients TROP2 low expression was associated with longer progression-free survival^30^. TROP2 knockdown mouse gastric dysplastic organoids showed limited growth and budding potential^31^. Nevertheless, in most cancers, high TROP2 levels were associated with tumor aggressiveness, metastasis and epithelial-to-mesenchymal transition^11,13,25,28^. TROP2 was detected in stem cells of different tissues and seems to be a stabilizer for the epithelial phenotype and self-renewal capacity. Correspondingly, Zhao et al. described an up-regulation of TROP2 upon *ZEB1* knockdown^32^. As a transmembrane glycoprotein TROP2 being in close interaction with other adhesion molecules such as claudins or integrins, it is involved in cell adhesion control. Huebner et al. showed a rather deadhesive phenotype in TROP2 high-expressing colon tumors^14^. Lenart et al. identified high TROP2 levels to maintain the epithelial barrier function in damaged lungs^33^. The different tissues and pathological conditions but also high intratumoral heterogeneity for TROP2 expression^14^ might be reasons for these divergent reports. Intratumoral heterogeneity might be caused by differences in methylation patterns in different tumor subclones. In this regard, it was shown that pre-treatment of metaplastic breast cancers with demethylating agent decitabine significantly improved the response to TROP2 ADCs^32^. Ultimately, the functions of TROP2 are highly controversial and need further investigation^28^.

In the present study, we add novel data about an inverse correlation between *TACSTD2* promoter methylation and the active H3K4me3 epigenetic mark in colon cancer cell lines and patients' tumor tissue. The in silico analysis revealed an increased association of the active H3K4me3 mark with the *TACSTD2* promoter when the tumor had high TROP2 expression. Moreover, this link was confirmed by immunohistochemical stainings in tumors with heterogeneous TROP2 expression reflecting such different tumor subpopulations.

An interplay between DNA methylation and histone modification has already been described^34^. In early embryogenesis, it was shown that DNA methylation can prevent H3K4 methylation, thus shaping chromatin structure^35^. However, histone lysine methylation in turn also affects DNA methylation^34^. The histone methyltransferases SET1A/B and MLL1/22/SETD1B play a key role in the methylation of histone 3 on lysine 4. This H3K4me3 mark was even shown to be mutually exclusive with DNA methylation^36,37^. One explanation could be that the binding of DNMT3B methyltransferase to the H3 tail is blocked when H3K4 is methylated^38^. To date, experimental data for different cancer types are still very rare.

## Conclusions

This study identified promoter methylation as an important factor in the regulation of TROP2 expression in colorectal cancer. Hypomethylation in concert with a higher association of the active histone code H3K4me3 at the *TACSTD2* promoter region led to an increased TROP2 protein expression. Whether TROP2-negative tumor cells with *TACSTD2* promoter methylation might present resistant cell populations under TROP2 inhibitor treatment with the TROP2-based ADC sacituzumab govitecan needs to be further investigated.

### Supplementary Information

Supplementary Information 1.

Supplementary Information 2.

Supplementary Figure 1.

Supplementary Figure 2.

Supplementary Figure 3.

Supplementary Figure 4.

Supplementary Figure 5.

Supplementary Figure 6.

Supplementary Figure 7.

Supplementary Table 1.

Supplementary Table 2.

Supplementary Table 3.

Supplementary Table 4.

## Data Availability

All ChIP-Seq data were downloaded from the GEO database under the accession number GSE143653. More experimental details are available upon request.

## Abbreviations

ADC: Antibody drug conjugate
AZA: 5-Azacytidine
Ctrl: Control
DNMT: DNA methyltransferase
DNMT1i: DNMT1 inhibitor
FBS: Fetal bovine serum
FFPE: Formalin-fixed, paraffin-embedded
H3K27: Lysine 27 of histone H3
H3K4me3: Trimethylated Lysine of histone H3
IHC: Immunohistochemical
MSP: Methylation-specific PCR
SDS-PAGE: SDS-polyacrylamide gel electrophoresis
TACSTD2: Tumor Associated Calcium Signal Transducer 2
TROP2: Trophoblast-Surface Antigen 2
