# Supplementary material for: DNA demethylation and tri-methylation of H3K4 at the TACSTD2 promoter are complementary players for TROP2 regulation in colorectal cancer cells
Source: Sci Rep. 2024 Feb 1;14:2683. doi: 10.1038/s41598-024-52437-1 (PMC10834991; doi:10.1038/s41598-024-52437-1)
Supplement: Supplementary file 1 — Supplementary Information 1. [file 41598_2024_52437_MOESM1_ESM.pdf]

# 1A

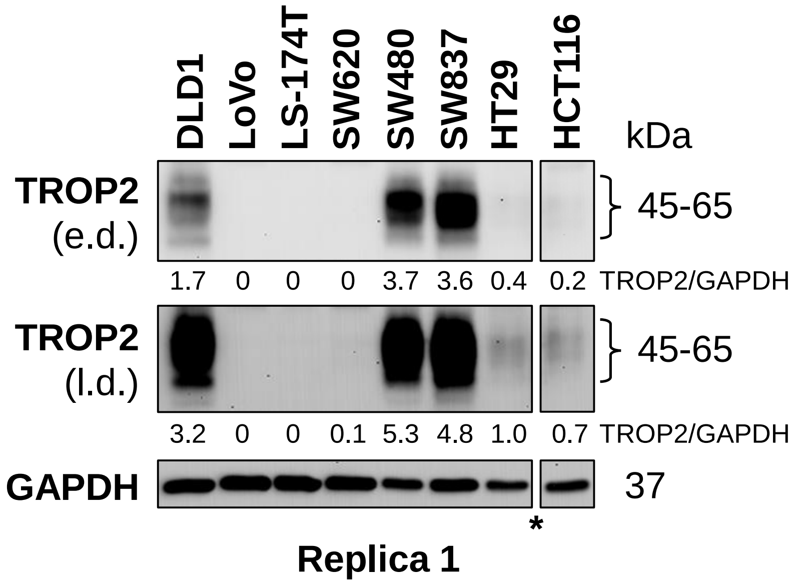


Short exposure

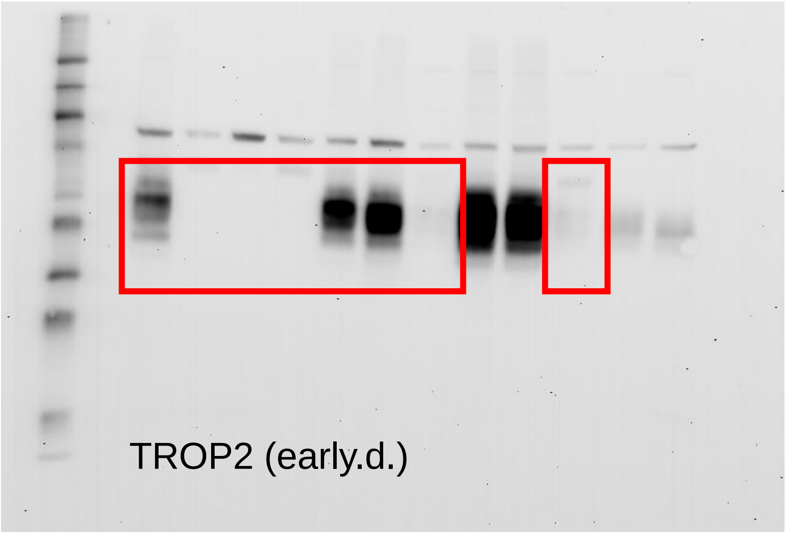


Long exposure

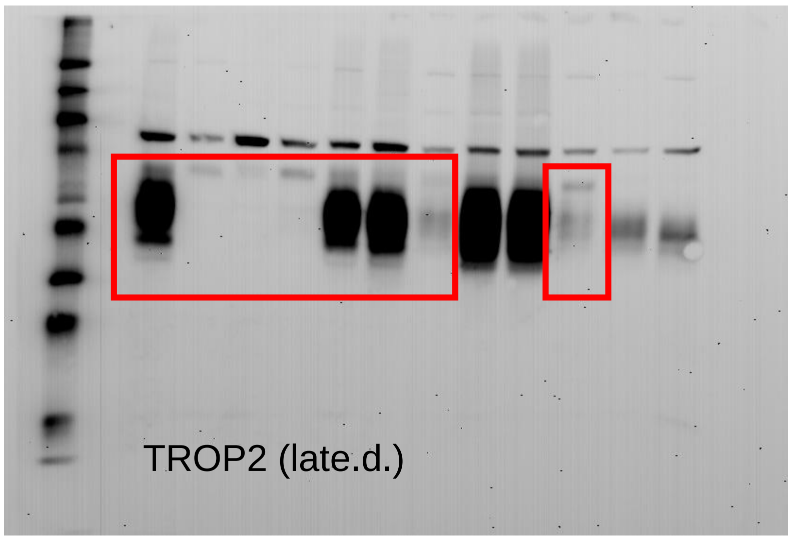


Both exposures are included in the manuscript

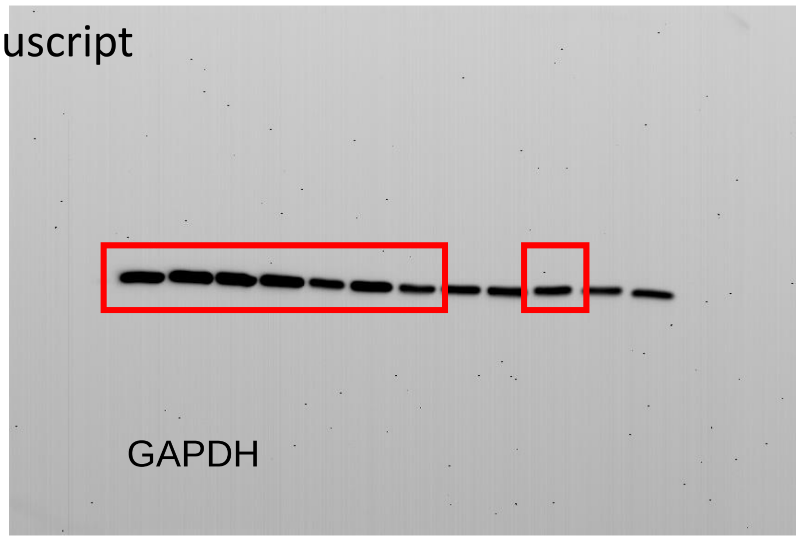

**1B**

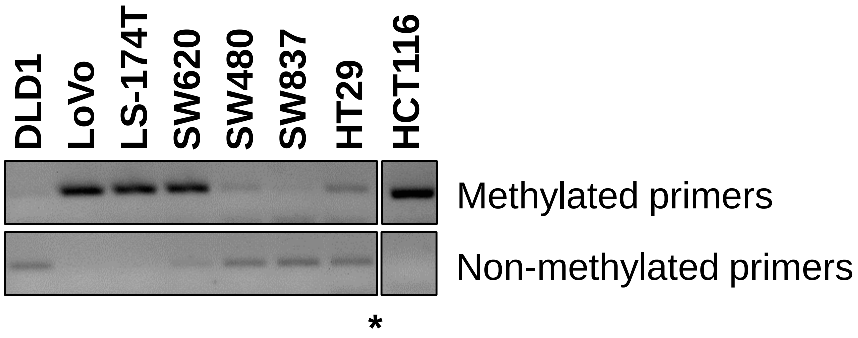


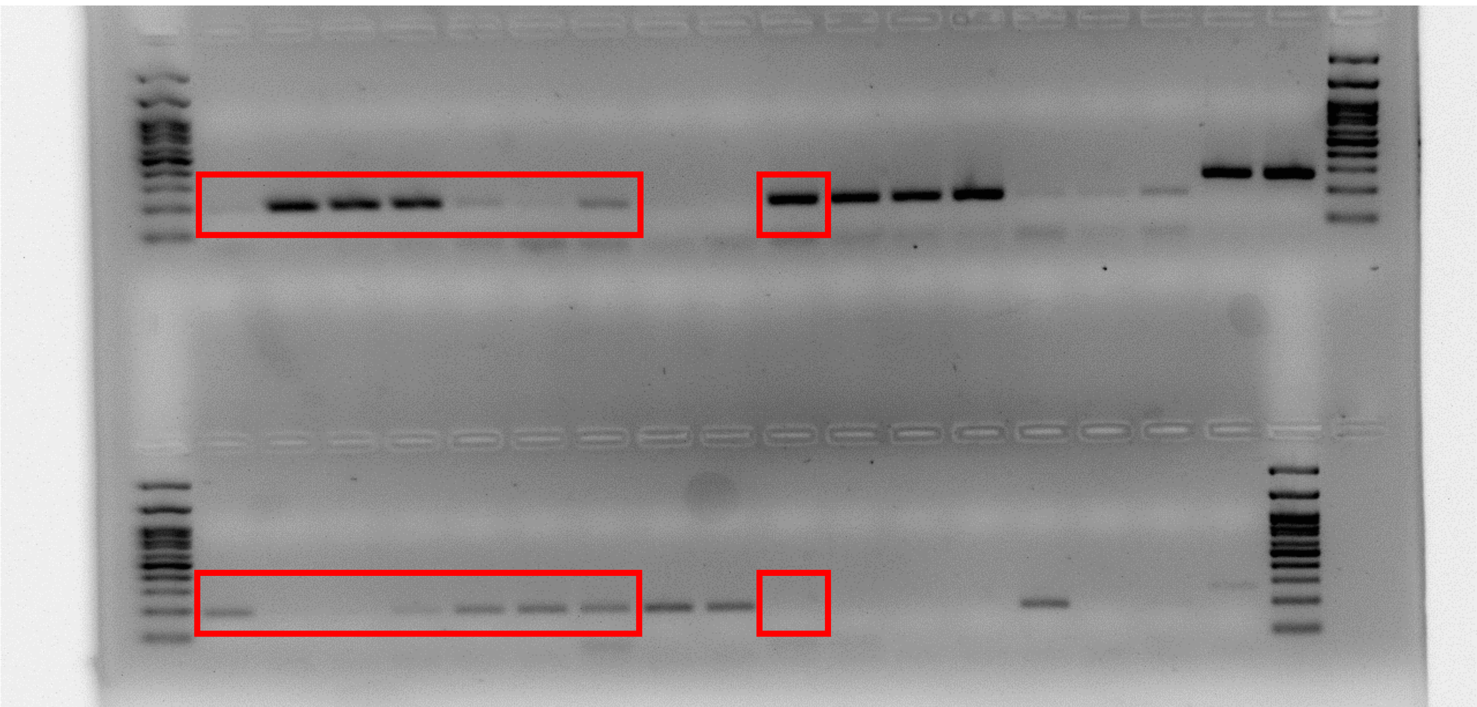

Agarose gels have been documented as a single photo made by UV camera
These images have been converted from the white signals on black background into black signal bands on gray background

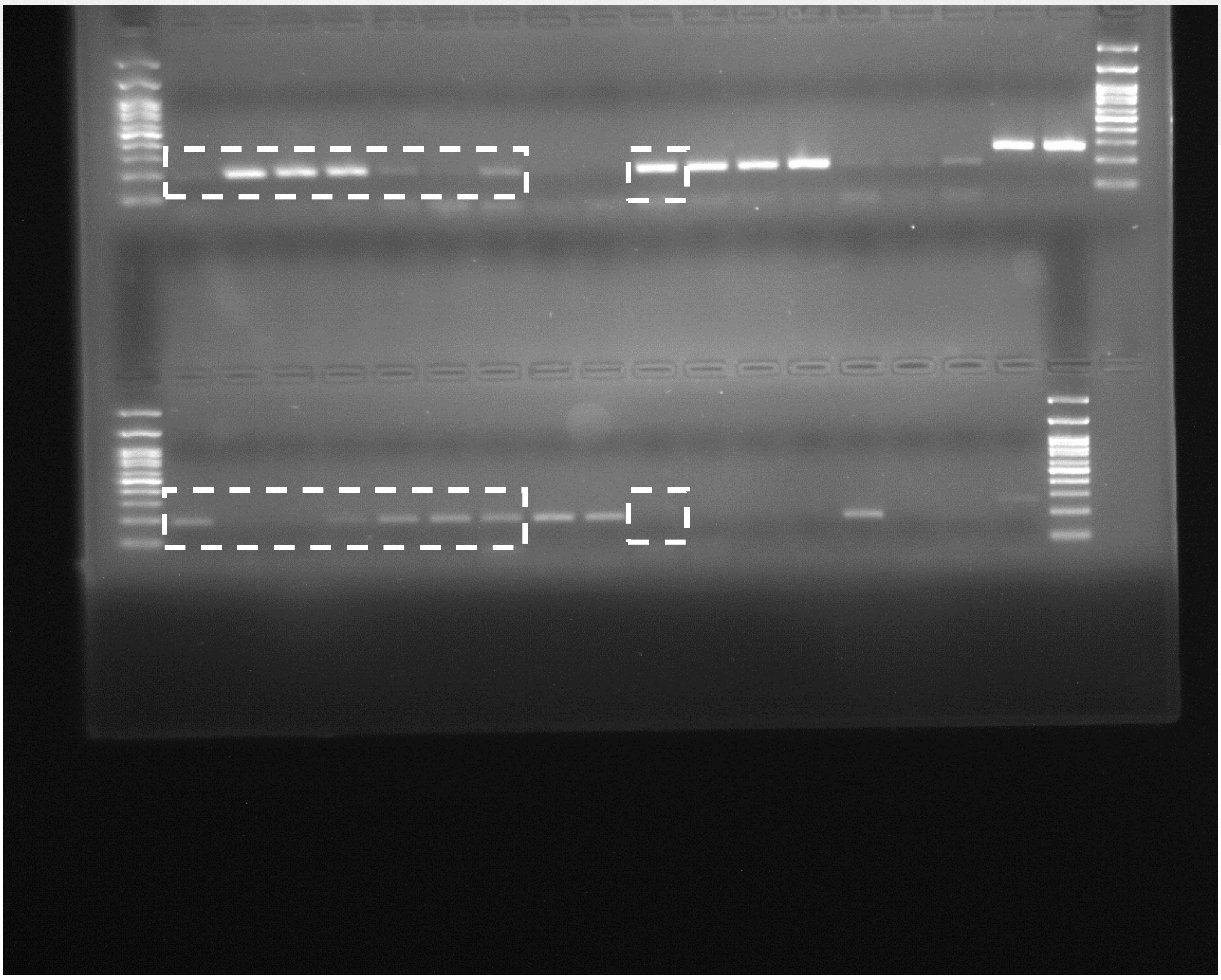

2C

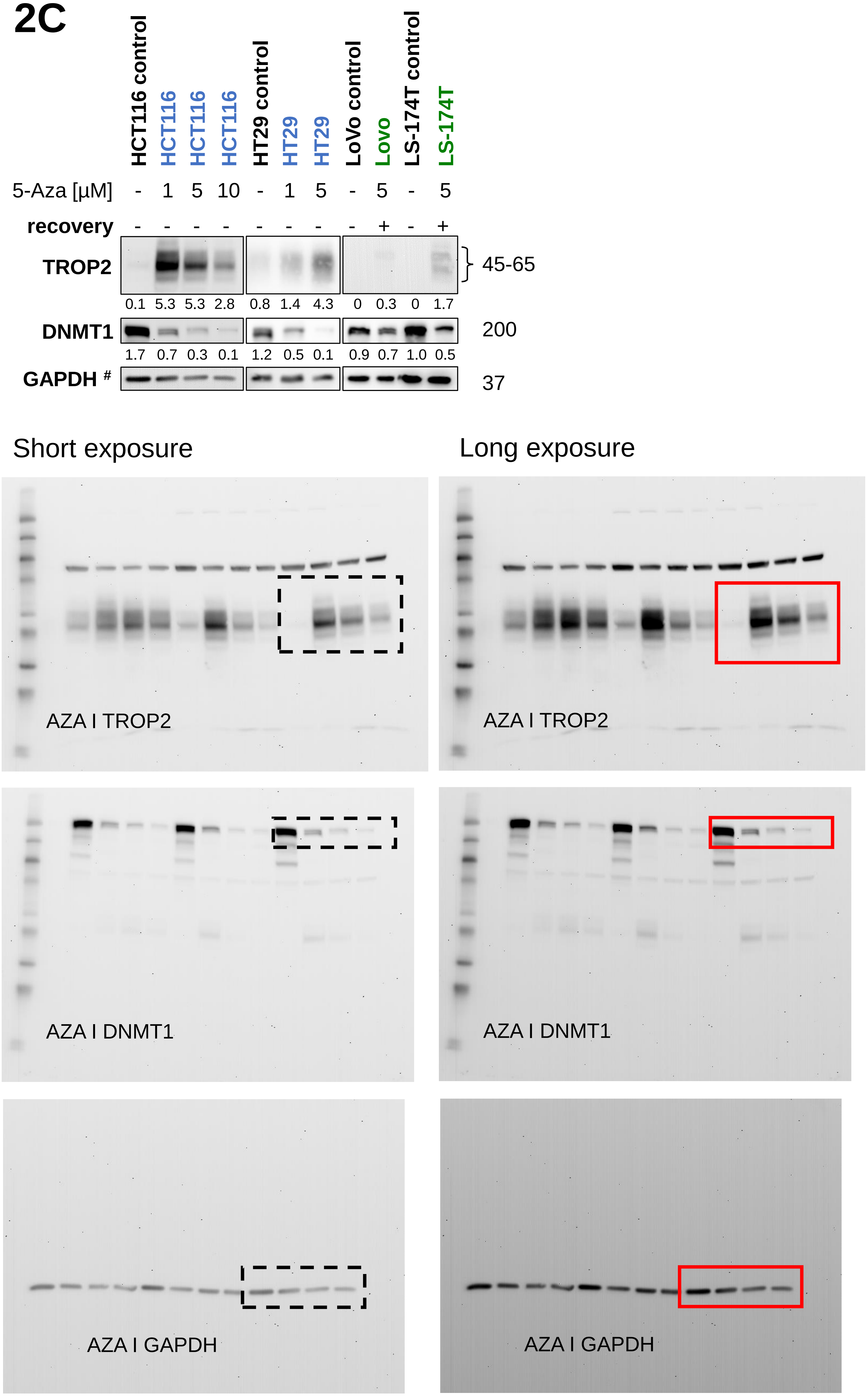
HCT116 control
HCT116
HCT116
HCT116
HT29 control
HT29
HT29
LoVo control
Lovo
LS-174T control
LS-174T
5-Aza [µM] - 1 5 10 - 1 5 - 5 - 5
recovery - - - - - - - - + - +
TROP2
45-65
0.1 5.3 5.3 2.8 0.8 1.4 4.3 0 0.3 0 1.7
DNMT1
200
1.7 0.7 0.3 0.1 1.2 0.5 0.1 0.9 0.7 1.0 0.5
GAPDH #
37
Short exposure
Long exposure
AZA I TROP2
AZA I TROP2
AZA I DNMT1
AZA I DNMT1
AZA I GAPDH
AZA I GAPDH

**2C**

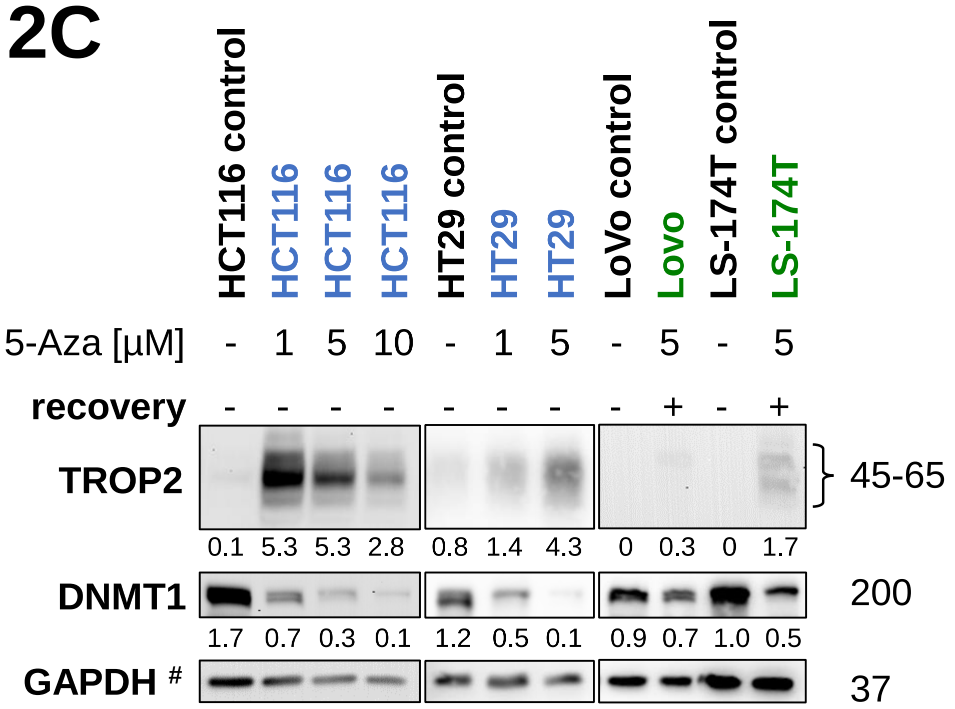


Short exposure

Long exposure

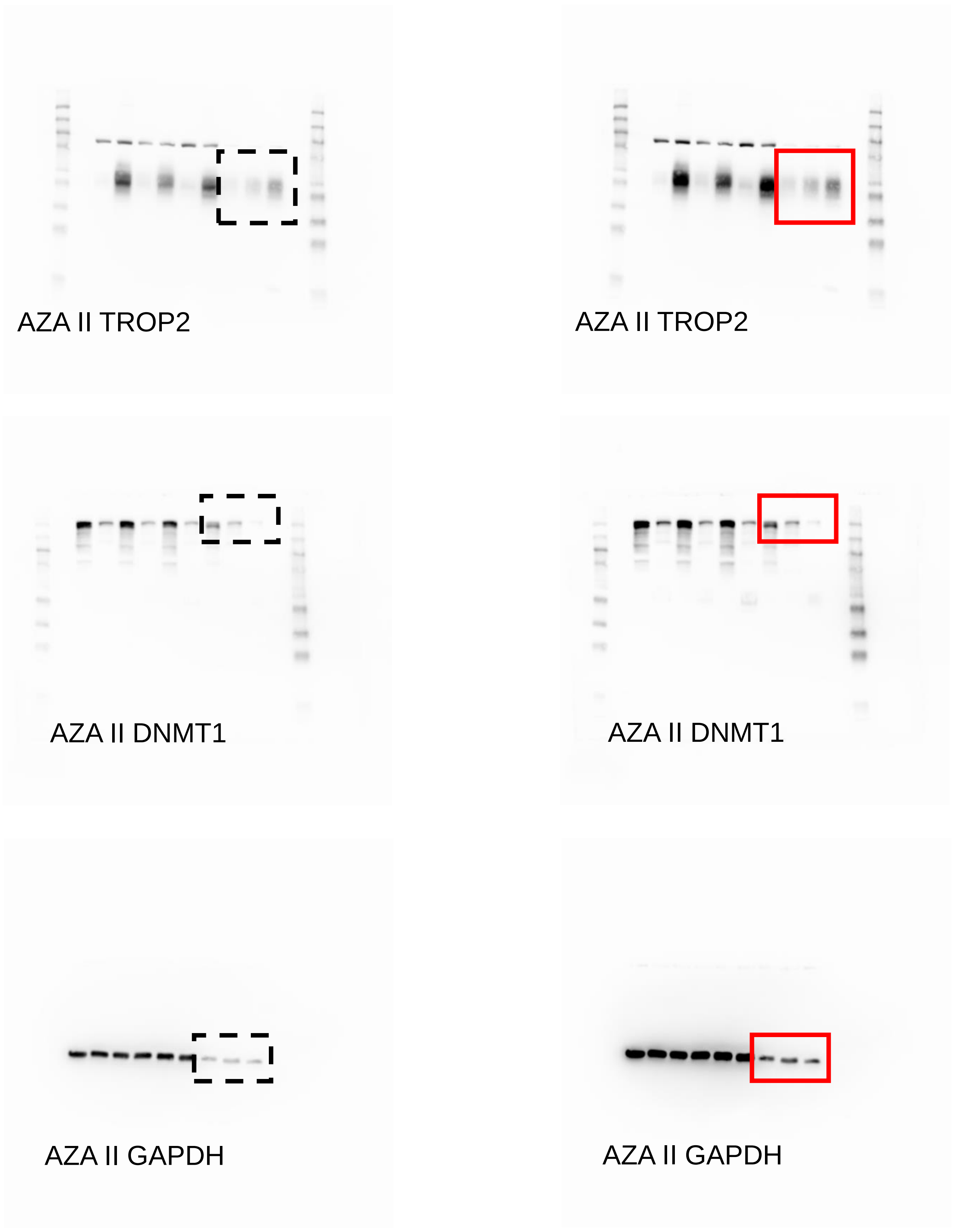

## 2C

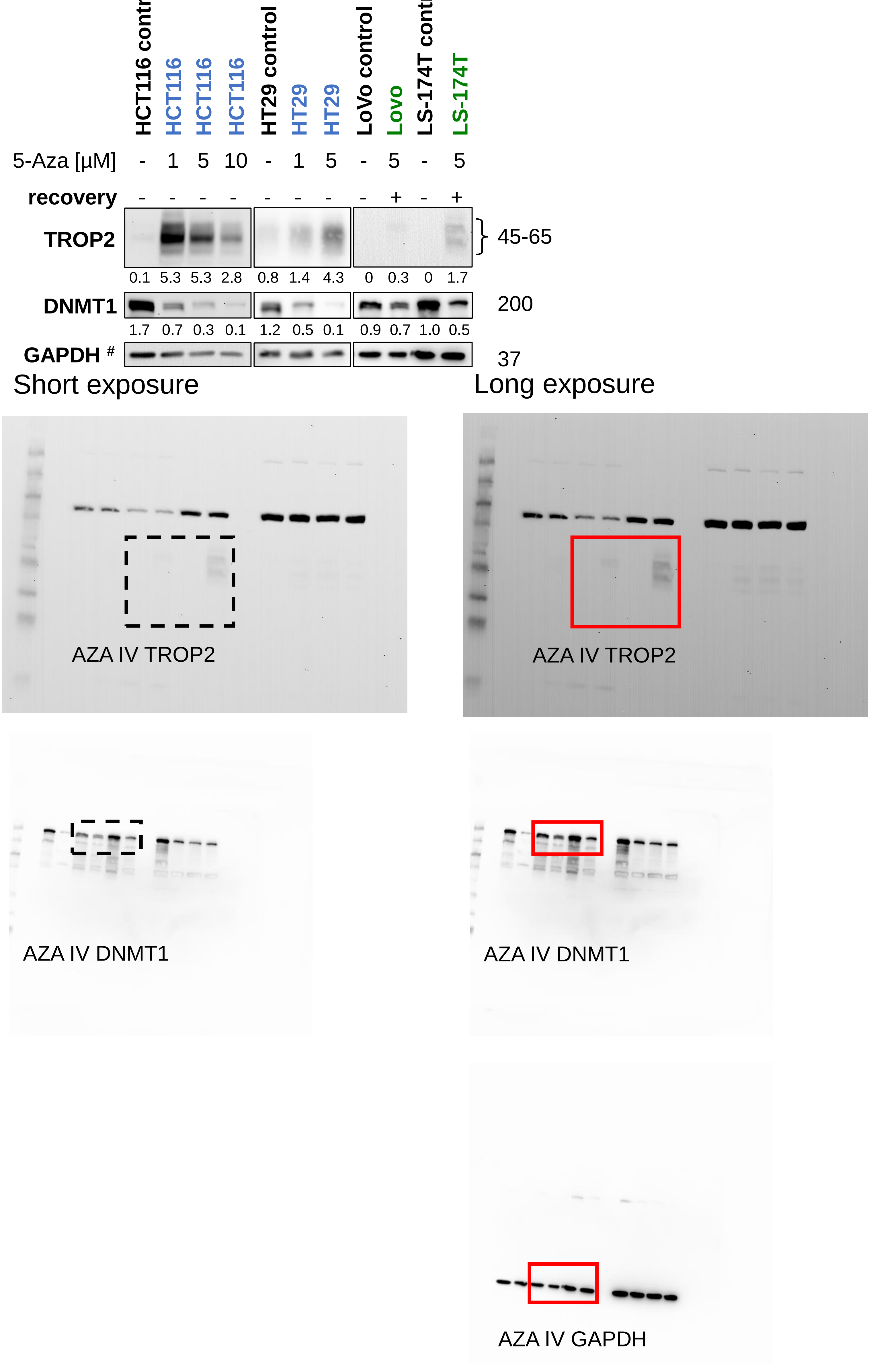
HCT116 control
HCT116
HCT116
HCT116
HT29 control
HT29
HT29
LoVo control
Lovo
LS-174T control
LS-174T
5-Aza [µM] - 1 5 10 - 1 5 - 5 - 5
recovery - - - - - - - - + - +
TROP2
45-65
0.1 5.3 5.3 2.8 0.8 1.4 4.3 0 0.3 0 1.7
DNMT1
200
1.7 0.7 0.3 0.1 1.2 0.5 0.1 0.9 0.7 1.0 0.5
GAPDH #
37
Short exposure
Long exposure
AZA IV TROP2
AZA IV TROP2
AZA IV DNMT1
AZA IV DNMT1
AZA IV GAPDH
