# Supplementary material for: DNA demethylation and tri-methylation of H3K4 at the TACSTD2 promoter are complementary players for TROP2 regulation in colorectal cancer cells
Source: Sci Rep. 2024 Feb 1;14:2683. doi: 10.1038/s41598-024-52437-1 (PMC10834991; doi:10.1038/s41598-024-52437-1)

Supplement Figure 2

A

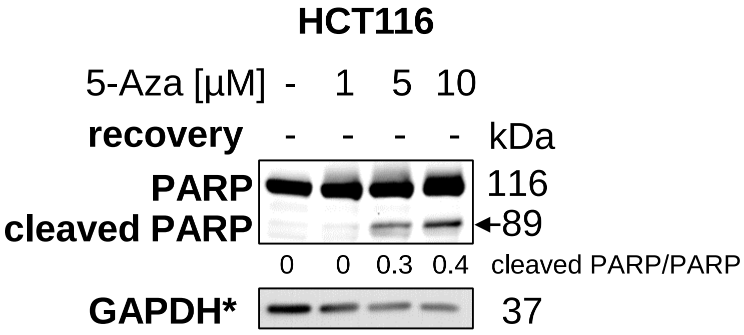

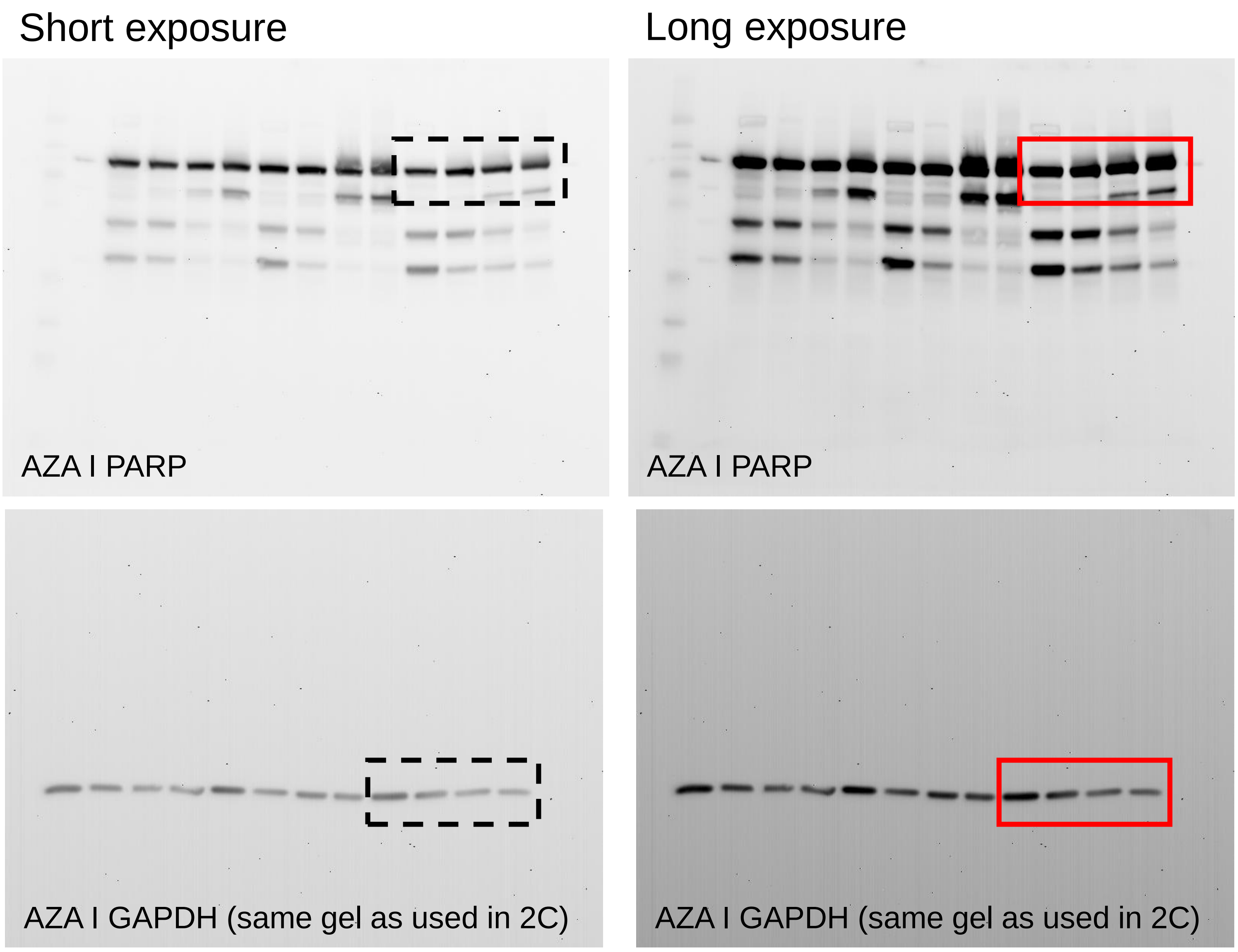

Supplement Figure 2

B

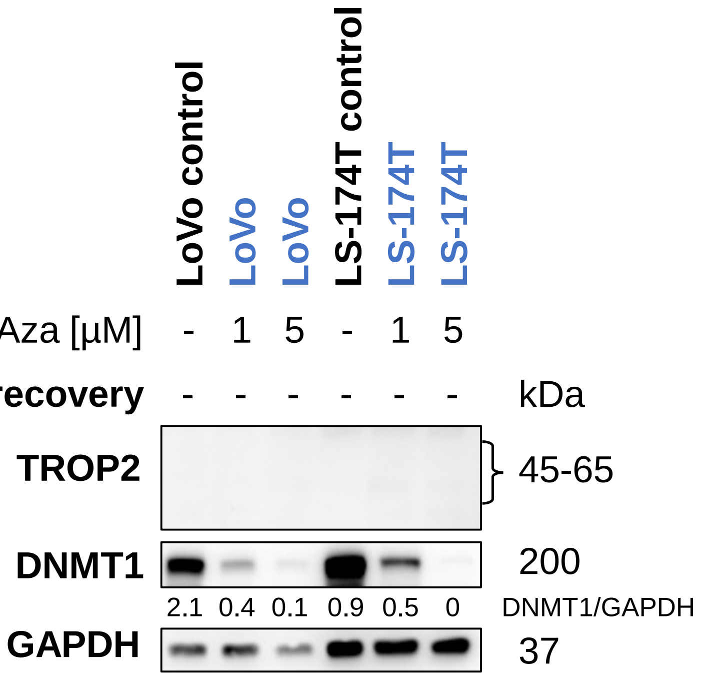

Short exposure

Long exposure

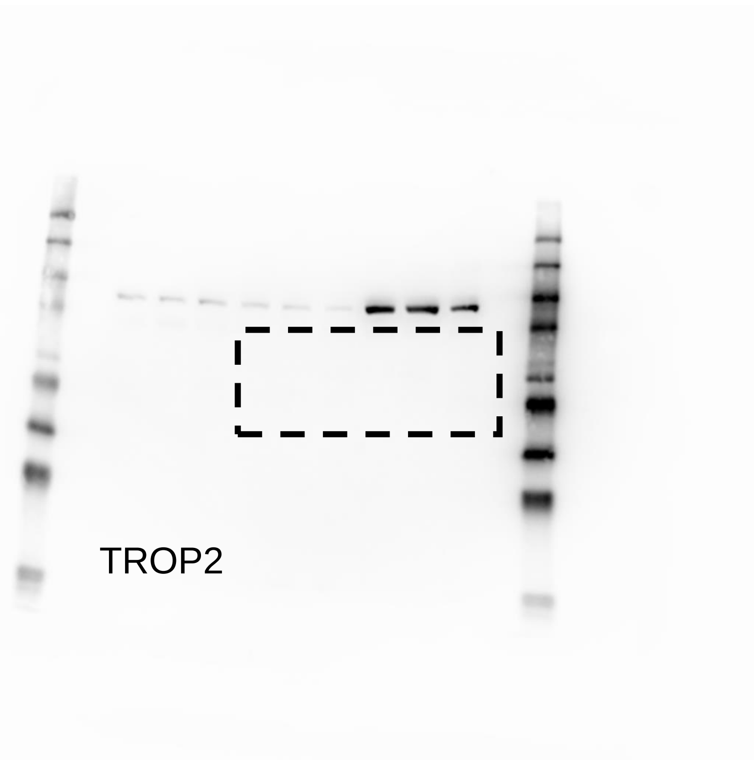

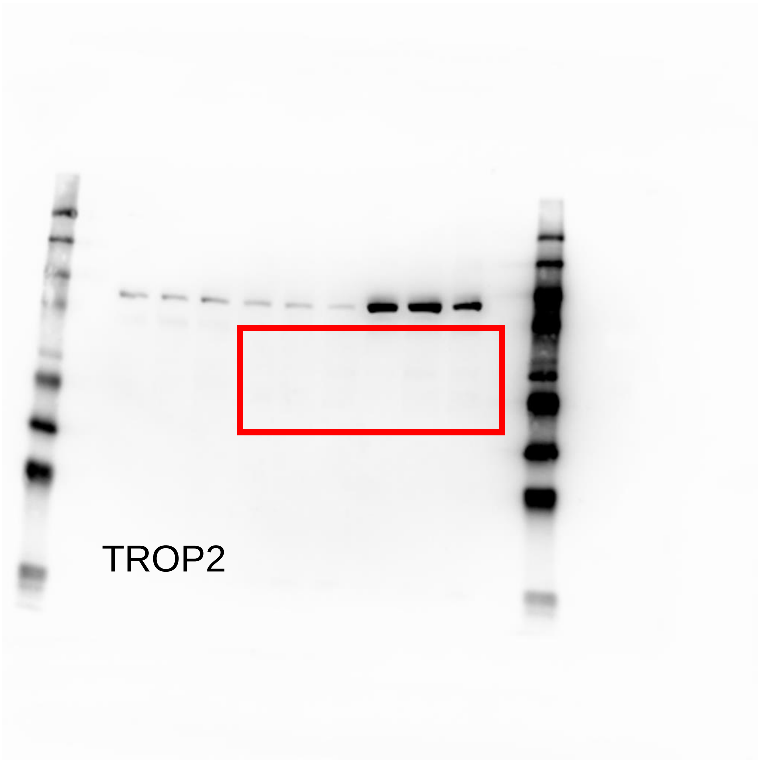

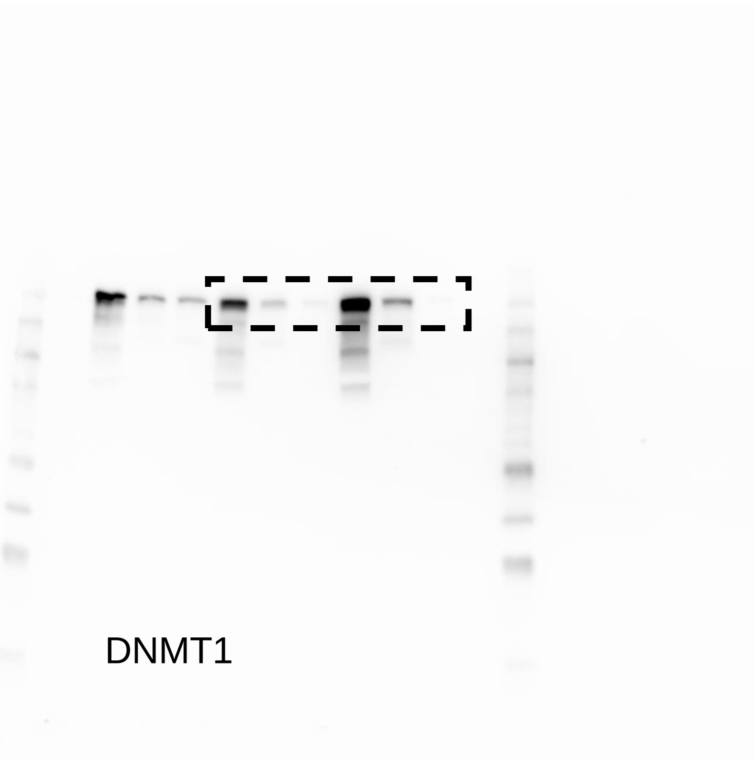

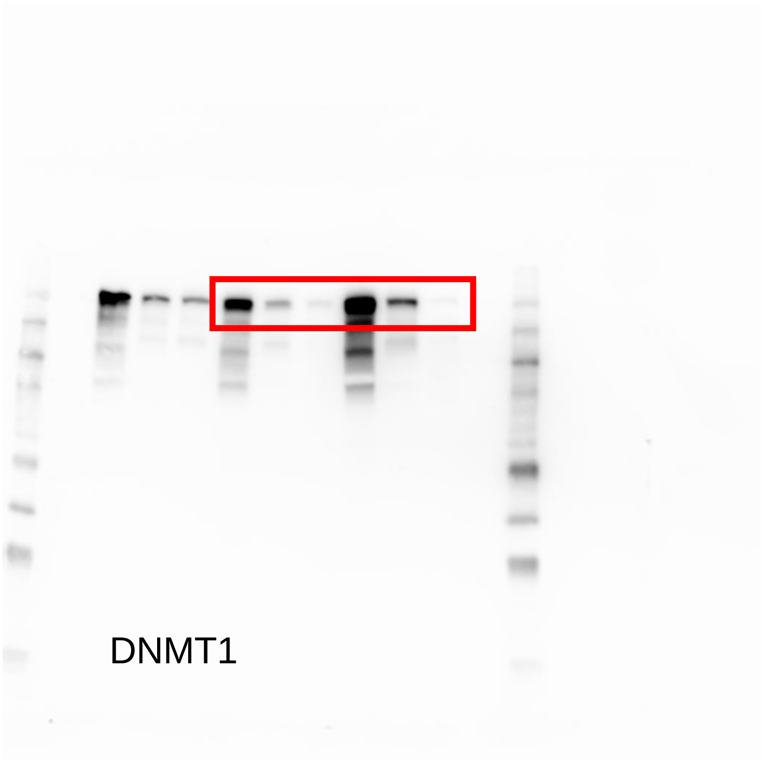

Supplement Figure 2

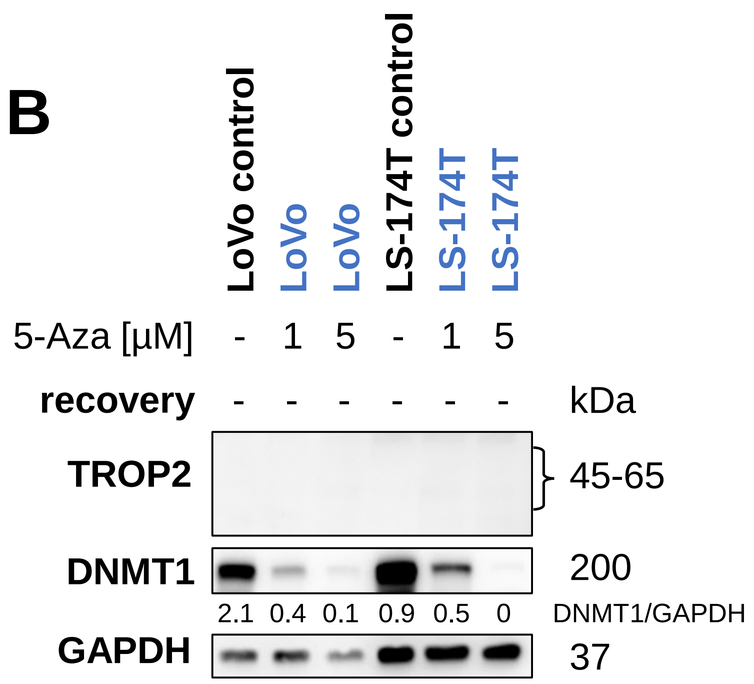

Short exposure

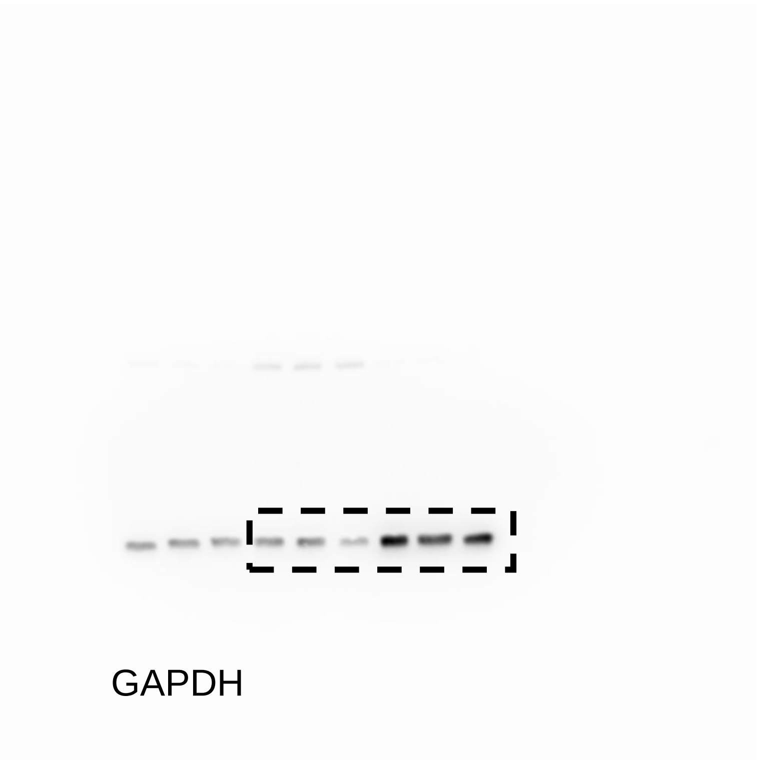

Long exposure

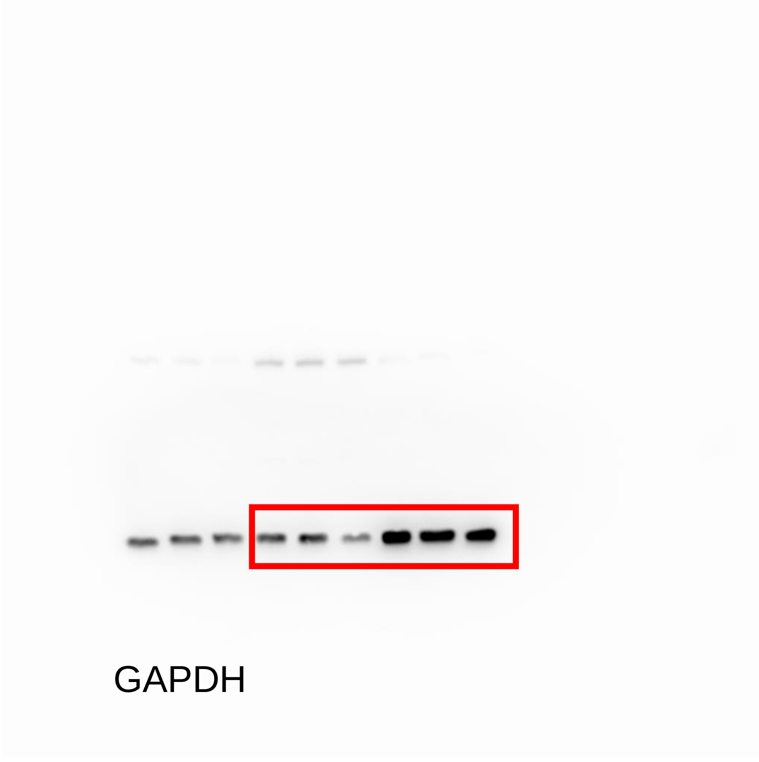

Supplement: Supplementary file 2 — Supplementary Information 2. [file 41598_2024_52437_MOESM2_ESM.pdf]
