# Supplementary material for: DNA demethylation and tri-methylation of H3K4 at the TACSTD2 promoter are complementary players for TROP2 regulation in colorectal cancer cells
Source: Sci Rep. 2024 Feb 1;14:2683. doi: 10.1038/s41598-024-52437-1 (PMC10834991; doi:10.1038/s41598-024-52437-1)
Supplement: Supplementary file 3 — Supplementary Figure 1. [file 41598_2024_52437_MOESM3_ESM.pdf]

A

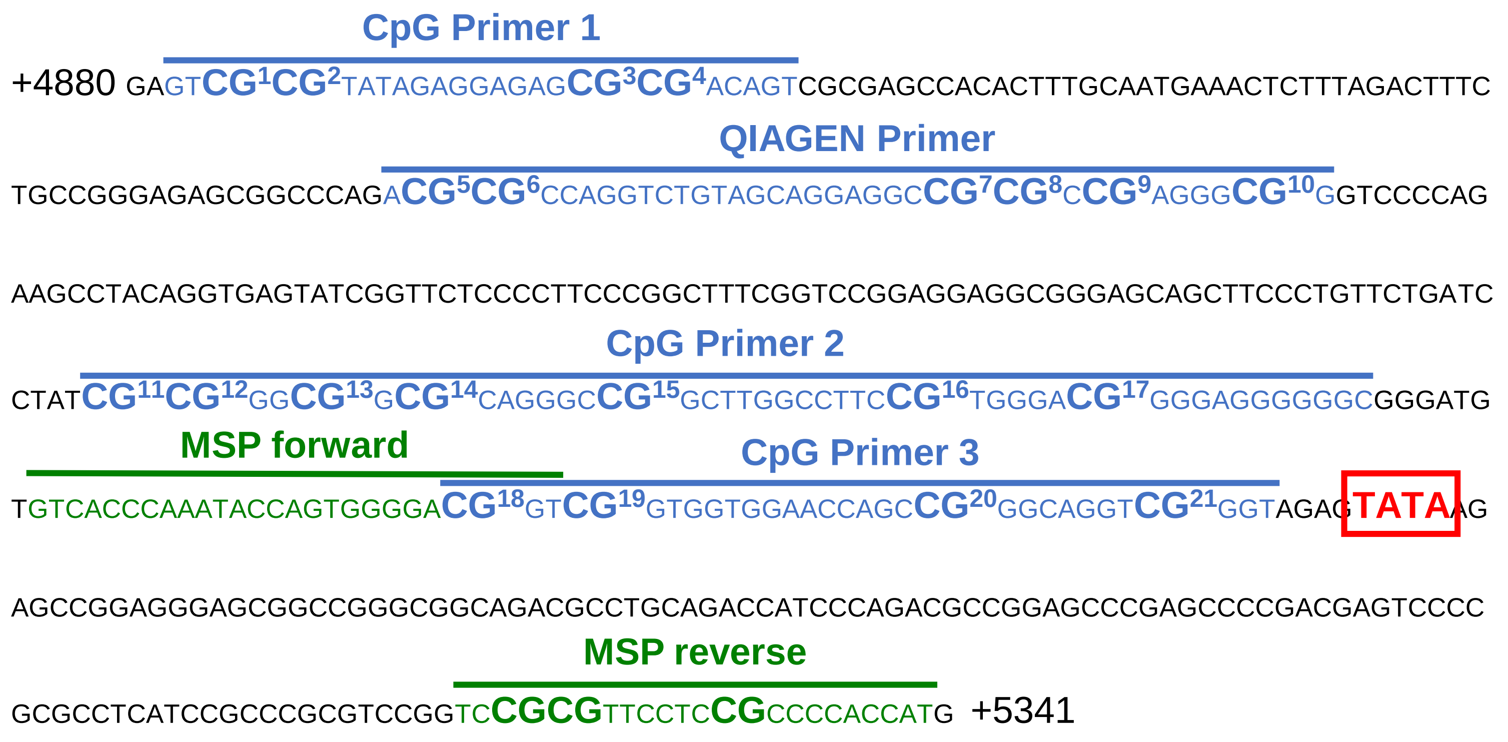

B

	forward	reverse	sequencing
**CpG primer 1**	5'-TAAAGAAGAGAGGGAG AGAGAA-3'	5'-ACCTCCTACTACAAACC T-3'	5'-GGAAAGAAAGAAAAGG G A-3'
**CpG primer 2**	5'-GGAGGAGGAGGGAGTA GTTTT-3'	5'-CCCCACTAATATTTAAAT AACACATCC-3'	5'-GGAGTAGTTTTTTTGTTT TGA-3'
**CpG primer 3**	5'-GGGGGGAGGGATGTGT TATTTAA-3'	5'-CTCCCTCCCACTCTTATA CTCTAC-3'	5'-GTGTTATTTAAATATTAGT GGGGA-3`

Supplementary Figure 1. Pyrosequencing. **(A)** *TACSTD2* promoter sequence with 21 CpG dinucleotides analysed via pyrosequencing with 4 primer pairs and 4 CpG dinucleotides analysed with one MSP primer pair. **(B)** Self-designed primer sequences for pyrosequencing.
