# Supplementary material for: DNA demethylation and tri-methylation of H3K4 at the TACSTD2 promoter are complementary players for TROP2 regulation in colorectal cancer cells
Source: Sci Rep. 2024 Feb 1;14:2683. doi: 10.1038/s41598-024-52437-1 (PMC10834991; doi:10.1038/s41598-024-52437-1)
Supplement: Supplementary file 4 — Supplementary Figure 2. [file 41598_2024_52437_MOESM4_ESM.pdf]

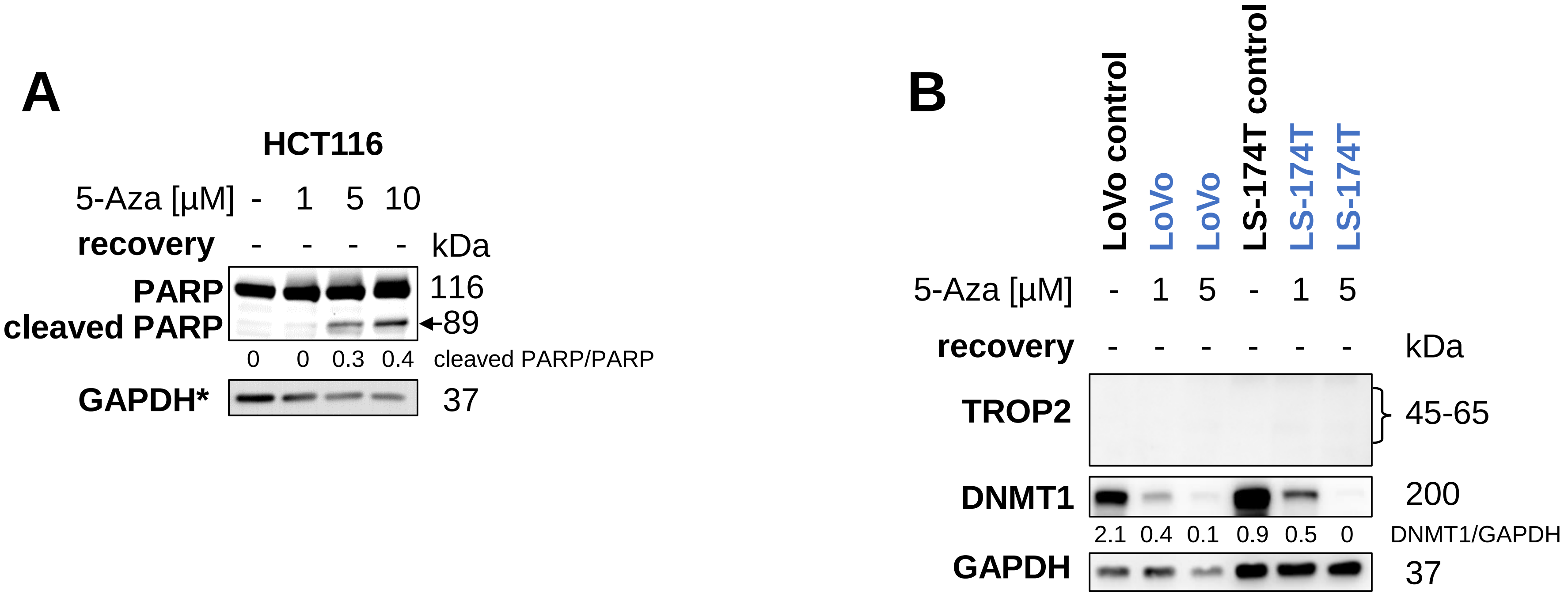

Supplementary Figure 2. (A) Cell death induced by 5-Azacytidine treatment quantified by PARP expression after treatment with different concentrations of 5-Azacytidine for HCT116 cell line, * the same GAPDH/same gel as used in Figure 2C. Ratios were calculated as cleaved PARP versus non-cleaved PARP (ImageJ). **(B)** TROP2 expression detected by Western blotting after treatment with DNMT1i in TROP2 non-expressing cell lines LoVo and LS-174T after 72 h of 5-Azacytidine treatment without an additional recovery phase. Band intensities were quantified using ImageJ analysis software, and ratios were calculated against the GAPDH band intensity.
