# Supplementary material for: DNA demethylation and tri-methylation of H3K4 at the TACSTD2 promoter are complementary players for TROP2 regulation in colorectal cancer cells
Source: Sci Rep. 2024 Feb 1;14:2683. doi: 10.1038/s41598-024-52437-1 (PMC10834991; doi:10.1038/s41598-024-52437-1)
Supplement: Supplementary file 5 — Supplementary Figure 3. [file 41598_2024_52437_MOESM5_ESM.pdf]

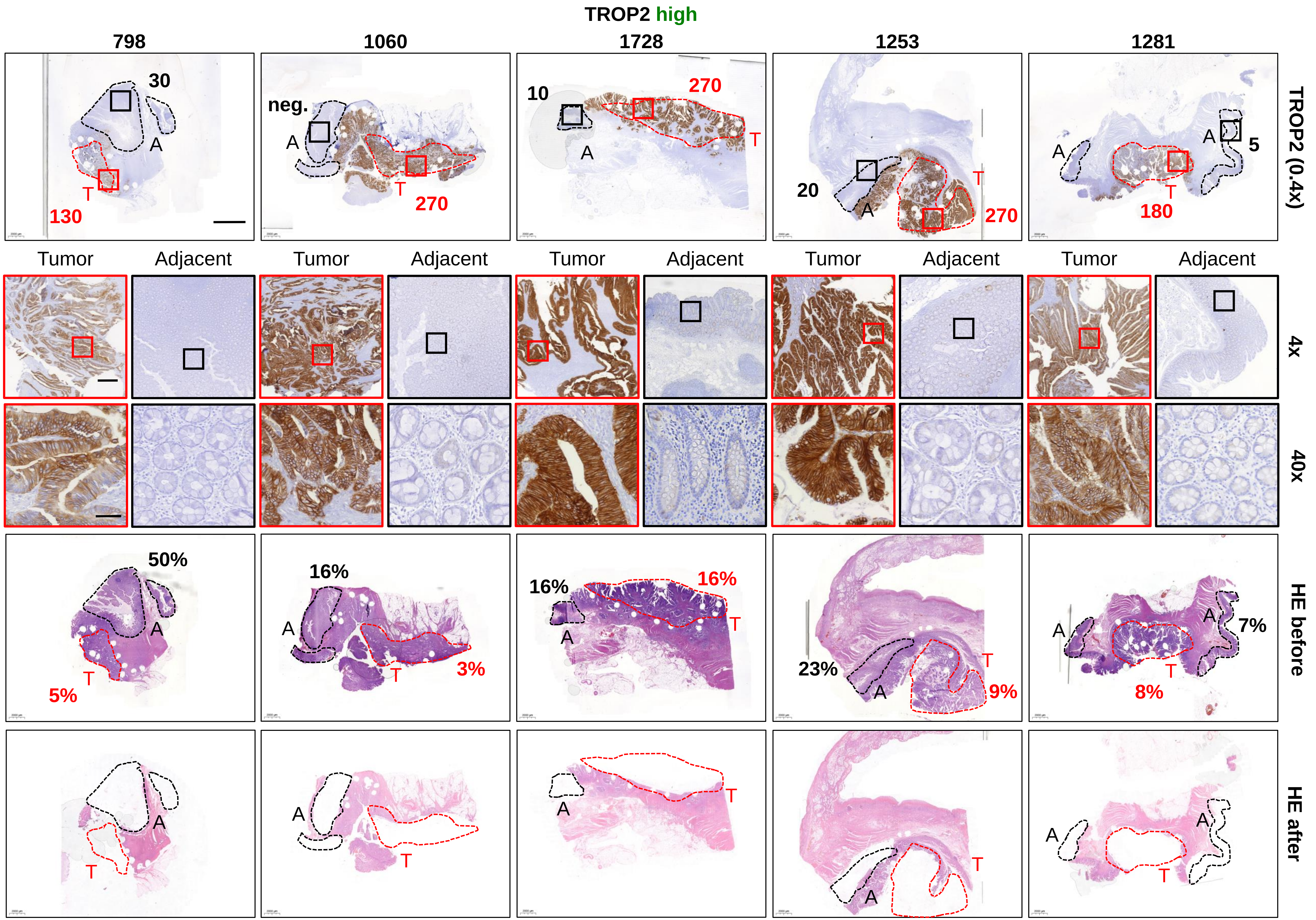

Supplementary Figure 3. Images of TROP2 high expressing tumors (TROP2 score >90). TROP2 IHC staining and HEs before and after scratching for DNA isolation. A=adjacent non-tumor (black), T=tumor (red). Marked sections match the scratched tumor and adjacent non-tumor areas for the assay, TROP2 score from IHC staining for tumor and adjacent non-tumor added in numbers into the TROP2 IHC stainingpictures, percentage in HEs shows average *TACSTD2* promoter methylation status for the different areas, scale bar in overview pictures equals 4000 µm, in the 4x magnification 400 µm and in 40x magnification 60 µm, *case 1060 is also shown in Figure 3A.
