# Supplementary material for: DNA demethylation and tri-methylation of H3K4 at the TACSTD2 promoter are complementary players for TROP2 regulation in colorectal cancer cells
Source: Sci Rep. 2024 Feb 1;14:2683. doi: 10.1038/s41598-024-52437-1 (PMC10834991; doi:10.1038/s41598-024-52437-1)
Supplement: Supplementary file 7 — Supplementary Figure 5. [file 41598_2024_52437_MOESM7_ESM.pdf]

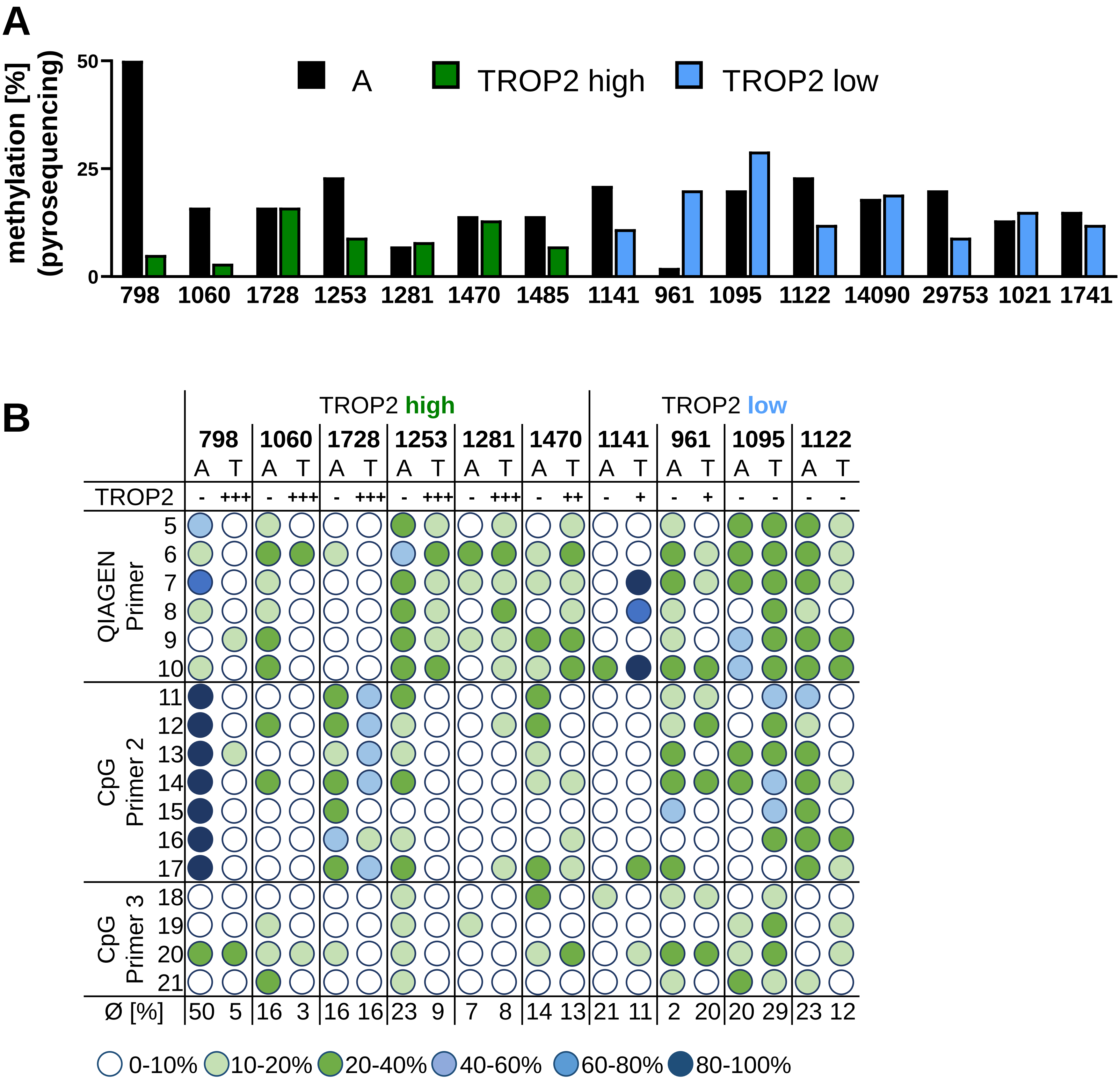

Supplementary Figure 5 (A) *TACSTD2* promoter methylation status compared in primary tumor (T) and adjacent non-tumor (A) samples: *TACSTD2* methylation [%] analysed by pyrosequencing for all cases divided into two groups using the median of the IHC score; TROP2 high expressing (TROP2 score >90) and TROP2 low expressing tumors (TROP2 score <90) **(B)** *TACSTD2* promoter methylation status of every single CpG site for exemplary ten cases, comparing tumor and adjacent non-tumor areas.
