# Supplementary material for: DNA demethylation and tri-methylation of H3K4 at the TACSTD2 promoter are complementary players for TROP2 regulation in colorectal cancer cells
Source: Sci Rep. 2024 Feb 1;14:2683. doi: 10.1038/s41598-024-52437-1 (PMC10834991; doi:10.1038/s41598-024-52437-1)
Supplement: Supplementary file 8 — Supplementary Figure 6. [file 41598_2024_52437_MOESM8_ESM.pdf]

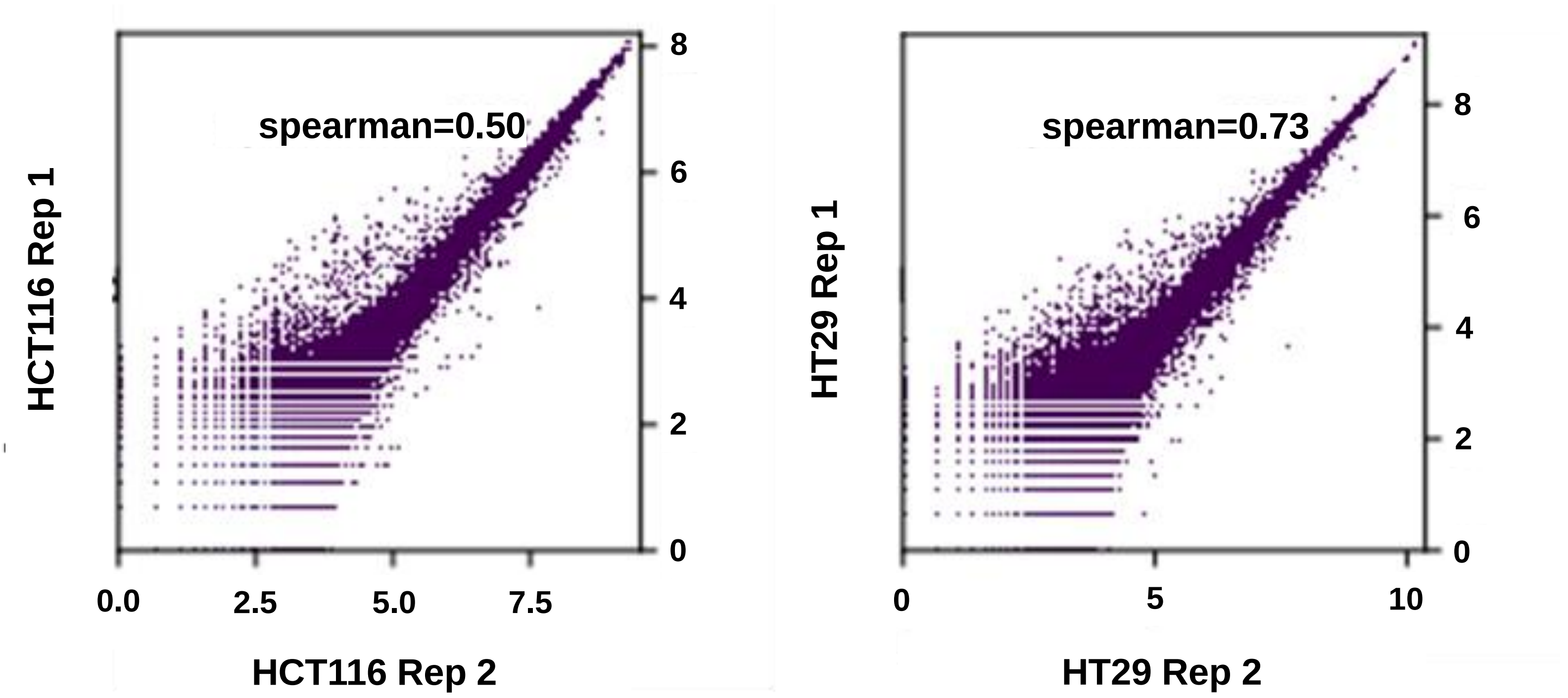

Supplementary Figure 6. Scatter plot representing the correlation between the biological replicates of H3K4me3 ChIP seq data in HCT116 and HT29 cell lines.
