# Supplementary material for: DNA demethylation and tri-methylation of H3K4 at the TACSTD2 promoter are complementary players for TROP2 regulation in colorectal cancer cells
Source: Sci Rep. 2024 Feb 1;14:2683. doi: 10.1038/s41598-024-52437-1 (PMC10834991; doi:10.1038/s41598-024-52437-1)
Supplement: Supplementary file 9 — Supplementary Figure 7. [file 41598_2024_52437_MOESM9_ESM.pdf]

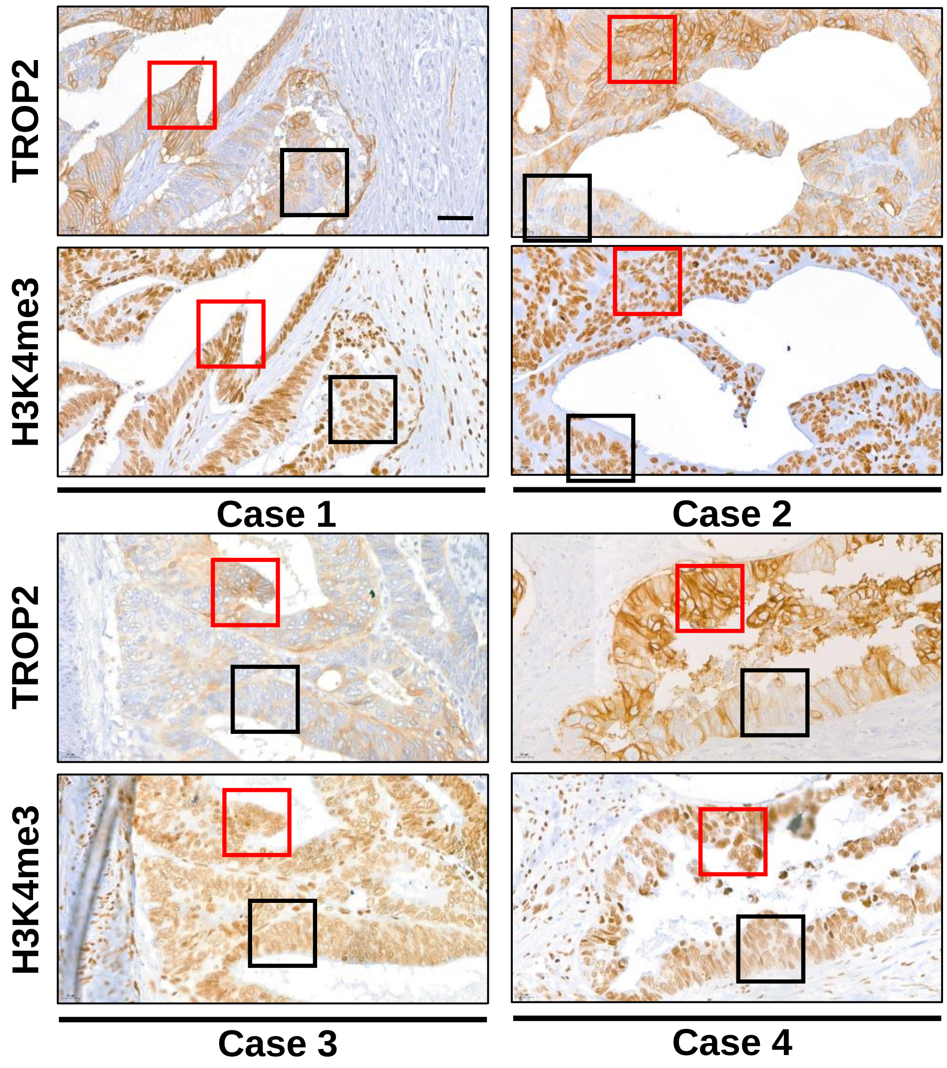

Supplementary Figure 7

Overview of intratumoral heterogeneity from the four TROP2 high tumor cases used in Figure 6A. Samples were stained for TROP2 and H3K4me3, red squares mark TROP2 high areas, black squares the TROP2 low areas, scale bar 40 µm.
