# Supplementary material for: DNA demethylation and tri-methylation of H3K4 at the TACSTD2 promoter are complementary players for TROP2 regulation in colorectal cancer cells
Source: Sci Rep. 2024 Feb 1;14:2683. doi: 10.1038/s41598-024-52437-1 (PMC10834991; doi:10.1038/s41598-024-52437-1)
Supplement: Supplementary file 10 — Supplementary Table 1. [file 41598_2024_52437_MOESM10_ESM.docx]

**Supplementary Table 1: Origin of cell lines**

| **Cell line** | **Tissue** | **Source** | **Catalogue number** |
| --- | --- | --- | --- |
| DLD1 | colon adenocarcinoma, human | Horizon Discovery, Cambridge, UK; | HD PAR-086 |
| HCT116 | colon adenocarcinoma, human | ATCC, Manassas, USA | CCL-247 |
| HT29 | colon adenocarcinoma, human | ATCC, Manassas, USA | HTB-38 |
| SW480 | colon adenocarcinoma, human | ATCC, Manassas, USA | CCL-228 |
| SW620 | lymph node metastasis, human | ATCC, Manassas, USA | CCL-227 |
| SW837 | rectal adenocarcinoma, human | Institute of Human Genetics, University Hospital Goettingen (Dr. Silke Keilfuß), Germany | n.a. |
| LoVo | colon adenocarcinoma, human | DSMZ - German Collection of Mircoorganisms and Cell Cultures GmbH, Germany | ACC 350 |
| LS-174T | colon adenocarcinoma, human | DSMZ - German Collection of Mircoorganisms and Cell Cultures GmbH, Germany | ACC 759 |
