# Supplementary material for: DNA demethylation and tri-methylation of H3K4 at the TACSTD2 promoter are complementary players for TROP2 regulation in colorectal cancer cells
Source: Sci Rep. 2024 Feb 1;14:2683. doi: 10.1038/s41598-024-52437-1 (PMC10834991; doi:10.1038/s41598-024-52437-1)
Supplement: Supplementary file 11 — Supplementary Table 2. [file 41598_2024_52437_MOESM11_ESM.docx]

**Supplementary Table 2**

**Patients clinicopathological data**

| **TROP2** | **age** | **sex** | **loc** | **M0/M1** | **pT** | **pN** | **stage** | **grading** | **lymph** | **vene** |
| --- | --- | --- | --- | --- | --- | --- | --- | --- | --- | --- |
| High | 68 | 2 | distal | 0 | 3 | 1 | III | 2 | 0 | 0 |
| High | 64 | 1 | distal | 1 | 4 | 0 | II | 3 | 1 | 0 |
| High | 90 | 2 | distal | 0 | 3 | 1 | III | 3 | 0 | 0 |
| High | 72 | 1 | distal | 0 | 2 | 0 | I | 1 | 0 | 0 |
| High | 55 | 2 | proximal | 0 | 2 | 0 | I | 1 | 1 | 1 |
| High | 75 | 2 | distal | 0 | 3 | 0 | II | 2 | 0 | 0 |
| High | 91 | 2 | rectal | 0 | 4 | 1 | III | 2 | 1 |  |
| High | 74 | 1 | rectal | 0 | 3 | 0 | II | 3 | 0 |  |
| High | 73 | 1 | rectal | 0 | 3 | 0 | II | 2 | 0 |  |
| High | 68 | 1 | distal | 0 | 3 | 0 | II | 2 | 0 | 0 |
| *High | 72 | 1 | proximal | 0 | 4 | 2 | III | 3 | 0 |  |
| *High | 74 | 2 | rectal | 1 | 2 | 1 | III | 2 | 1 |  |
| Low | 63 | 2 | distal | 0 | 3 | 1 | III | 2 | 1 | 0 |
| Low | 72 | 2 | proximal | 0 | 2 | 0 | I | 3 | 0 | 1 |
| Low | 68 | 2 | distal | 0 | 2 | 0 | I | 2 | 0 | 0 |
| Low | 64 | 2 | proximal | 0 | 1 | 0 | I | 2 | 0 | 0 |
| Low | 84 | 1 | rectal | 0 | 3 | 0 | II | 3 | 0 |  |
| Low | 57 | 2 | rectal | 0 | 2 | 0 | I | 2 | 0 |  |
| Low | 63 | 1 | rectal | 0 | 3 | 1 | III | 3 | 1 |  |
| Low | 49 | 1 | rectal | 0 | 3 | 1 | III | 2 | 1 |  |
| Low | 75 | 1 | rectal | 0 | 2 | 0 | I | 2 | 0 |  |
| Low | 70 | 2 | rectal | 0 | 3 | 0 | II | 2 | 0 |  |
| Low | 79 | 2 | rectal | 0 | 3 | 1 | III | 2 | 0 |  |
| Low | 70 | 1 | rectal | 0 | 3 | 0 | II | 2 | 0 |  |
| Low | 54 | 1 | rectal | 0 | 3 | 0 | II | 2 | 0 |  |
| Low | 70 | 2 | proximal | 0 | 2 | 0 | I | 3 | 0 | 0 |
| Low | 68 | 1 | distal | 1 | 2 | 0 | I | 2 | 0 | 0 |
| *Low | 71 | 2 | proximal | 0 | 2 | 0 | I | 3 | 0 |  |
| *Low | 62 | 2 | rectal | 0 | 3 | 0 | II | 2 | 0 |  |
| *Low | 75 | 1 | proximal | 0 | 3 | 1 | I | 3 | 0 |  |
| *Low | 76 | 1 | distal | 0 | 3 | 0 | II | 2 | 0 |  |
| *Low | 50 | 1 | rectal | 0 | 2 | 2 | III | 3 | 1 |  |
| *Low | 70 | 1 | rectal | 1 | 2 | 0 | I | 2 | 0 |  |

loc – localization

*only normal (non-adjacent) tissue used
