# Supplementary material for: DNA demethylation and tri-methylation of H3K4 at the TACSTD2 promoter are complementary players for TROP2 regulation in colorectal cancer cells
Source: Sci Rep. 2024 Feb 1;14:2683. doi: 10.1038/s41598-024-52437-1 (PMC10834991; doi:10.1038/s41598-024-52437-1)
Supplement: Supplementary file 12 — Supplementary Table 3. [file 41598_2024_52437_MOESM12_ESM.docx]

**Supplementary Table 3: Antibodies used for Western Blots (experimental details)**

| **Primary antibody** | **Dilution** | **Molecular weight (kDa)** | **Manufacturer** |
| --- | --- | --- | --- |
| TROP2 (D1W5W)  Rabbit IgG | 1:2.000 | 45-65 | Cell Signaling Technologies, USA |
| DNMT1  Rabbit IgG | 1:1.000 | 200 | Cell Signaling Technologies, USA |
| PARP (46D11)  Rabbit IgG | 1:2.000 | 89, 116 | Cell Signaling Technologies, USA |
| GAPDH (6C5) | 1:100.000 | 37 | Abnova, USA |
| **Secondary Antibody** | **Dilution** | **Molecular weight (kDa)** | **Manufacturer** |
| Anti-biotin | 1:5.000 | / | Cell Signaling Technologies, USA |
