# Supplementary material for: DNA demethylation and tri-methylation of H3K4 at the TACSTD2 promoter are complementary players for TROP2 regulation in colorectal cancer cells
Source: Sci Rep. 2024 Feb 1;14:2683. doi: 10.1038/s41598-024-52437-1 (PMC10834991; doi:10.1038/s41598-024-52437-1)
Supplement: Supplementary file 13 — Supplementary Table 4. [file 41598_2024_52437_MOESM13_ESM.docx]

**Supplementary Table 4**

**Clinicopathological data from ChIP seq/RNA seq data set (GSE156613)**

| **TROP2** | **sex** | **M0/M1** | **pT** | **pN** | **TNM stage** | **Pathological diagnosis** |
| --- | --- | --- | --- | --- | --- | --- |
| High | 1 | 0 | 4 | 2 | T4aN2bM0 | Poorly differentiated rectal adenocarcinoma |
| High | 2 |  |  |  |  | Moderately differentiated descending colon adenocarcinoma |
| High | 2 |  |  |  |  | Moderately differentiated colon adenocarcinoma |
| Low | 2 | 0 | 4 | 0 | T4N0M0 | Poorly differentiated adenocarcinoma |
| Low | 1 |  |  |  |  | Moderately differentiated adenocarcinoma of splenic flexure of colon |
| Low | 2 | 0 | 3 | 0 | T3N0M0 | Moderately differentiated colon adenocarcinoma |
